# Fundamental interplay between anionic/cationic redox governing the kinetics and thermodynamics of lithium-rich cathodes

**DOI:** 10.1038/s41467-017-02291-9

**Published:** 2017-12-20

**Authors:** Gaurav Assat, Dominique Foix, Charles Delacourt, Antonella Iadecola, Rémi Dedryvère, Jean-Marie Tarascon

**Affiliations:** 1 Collège de France, Chimie du Solide et de l’Energie—UMR CNRS 8260, 11 Place Marcelin Berthelot, 75005 Paris, France; 2 Réseau sur le Stockage Electrochimique de l’Energie (RS2E)—FR CNRS 3459, 80039 Amiens Cedex, France; 3 UPMC Univ Paris 06, Sorbonne Universités, 4 Place Jussieu, 75005 Paris, France; 4 IPREM—UMR 5254 CNRS, Université de Pau et des Pays de l’Adour, Hélioparc, 2 Avenue Pierre Angot, 64053 Pau Cedex 9, France; 5 Laboratoire de Réactivité et Chimie des Solides (LRCS)—UMR CNRS 7314, Université de Picardie Jules Verne, 33 rue Saint Leu, 80039 Amiens Cedex, France

## Abstract

Reversible anionic redox has rejuvenated the search for high-capacity lithium-ion battery cathodes. Real-world success necessitates the holistic mastering of this electrochemistry’s kinetics, thermodynamics, and stability. Here we prove oxygen redox reactivity in the archetypical lithium- and manganese-rich layered cathodes through bulk-sensitive synchrotron-based spectroscopies, and elucidate their complete anionic/cationic charge-compensation mechanism. Furthermore, via various electroanalytical methods, we answer how the anionic/cationic interplay governs application-wise important issues—namely sluggish kinetics, large hysteresis, and voltage fade—that afflict these promising cathodes despite widespread industrial and academic efforts. We find that cationic redox is kinetically fast and without hysteresis unlike sluggish anions, which furthermore show different oxidation vs. reduction potentials. Additionally, more time spent with fully oxidized oxygen promotes voltage fade. These fundamental insights about anionic redox are indispensable for improving lithium-rich cathodes. Moreover, our methodology provides guidelines for assessing the merits of existing and future anionic redox-based high-energy cathodes, which are being discovered rapidly.

## Abstract (web summary)

Anionic redox chemistry has enabled the design of high-capacity battery cathodes for energy storage. Here, the authors demonstrate reversible anionic redox in an archetypical lithium-rich oxide via bulk-sensitive spectroscopies, further revealing its crucial role in practically important properties.

## Introduction

Rechargeable batteries are enabling the widespread adoption of electrified transportation and renewable energy. System-level predictions reveal that Li-ion batteries powered by Li-rich layered-oxide cathodes, e.g., Li_1.2_Ni_0.13_Mn_0.54_Co_0.13_O_2_ (LR-NMC), hold the highest promise regarding practical energy density and cost.^1,2^ LR-NMC can deliver capacities reaching 300 mAh g^−1^, a value under-explained if solely the transition metals (TMs) participate in redox processes. Recent advances have steered a consensus that such high capacities arise from the reversible redox of O^2–^ anions.^3–5^ Anionic redox has thereby emerged as a transformational approach for designing new high-energy cathodes, several of which have been discovered lately with diverse crystal chemistries.^6,7^

As battery researchers are moving into this new direction, it is necessary to revisit the Li-rich systems with a fresh perspective by including anionic redox in order to understand the fundamental origins of some practical roadblocks (i.e., voltage fade, poor kinetics, and voltage hysteresis). These issues jeopardize cycle life, power rate, energy efficiency, and state of charge (SoC) management, and hence have plagued the commercialization of LR-NMCs despite several years of academic and industrial interest.^8,9^ Recently, we pointed out the poor electrochemical kinetics of anionic redox and its detrimental role in triggering voltage fade and hysteresis using a “model” Li-rich layered-oxide, Li_2_Ru_0.75_Sn_0.25_O_3_.^10^ In light of these findings, it is imperative to ask whether the same is true for LR-NMC since it is an archetypical Li-rich system of substantial real-world potential.

This task is not straightforward because LR-NMC is complicated by the redox activity of Ni, Mn, Co, and O, whose redox potentials must first be identified to establish the charge-compensation mechanism. Starting with oxygen, it was believed for quite a few years that the anomalous first charge capacity is compensated by irreversible oxygen loss from the electrode’s surface resulting in densification.^11–14^ However, recent measurements suggest that such oxygen loss is largely insufficient to account for the extra first charge capacity.^4,15,16^ Thus, the early models of surface oxygen loss were only partially complete and it is now believed that most of the oxidized oxygen remains in the lattice and participates in redox^17,18^, as directly observed via ex situ X-ray absorption spectroscopy (XAS)^4,19,20^, X-ray photoelectron spectroscopy (XPS)^21^, and hard X-ray photoelectron spectroscopy (HAXPES)^22^. The remaining task now is to look beyond the first charge and identify the electrochemical potentials with corresponding states of charge (SoC) where anionic redox occurs.

As for charge compensation from the TMs, operando XAS^23–25^ as well as ex situ XAS^4,20,26–28^ at TM K-edges have clearly shown how the Ni^2+/3+/4+^ redox process proceeds but there are still discrepancies concerning the extent of Mn^3+/4+^ contribution because Mn K-edge interpretation is not unambiguous. Finally for Co, the XAS data in literature is insufficient to precisely conclude the redox potential of Co^3+/4+^, which is often wrongly assumed when interpreting the differential capacity (d*Q*/d*V*) plots. We note that soft-XAS at Mn and Co L-edges can provide a clearer signature of oxidation states, but these data are scarce in literature.^19,29,30^

Filling the above-mentioned charge-compensation knowledge gaps is necessary to explore the role of anionic redox on battery performance issues, i.e., voltage hysteresis, poor kinetics, and voltage fade. So far, the Mn-rich nature of LR-NMC leaning toward Li_2_MnO_3_ was blamed for these issues. A phenomenological model involving irreversible and reversible TM migrations was proposed to explain voltage fade and hysteresis, respectively.^31^ However, it cannot be ruled out that such migrations are in fact a consequence of anionic redox. Concerning kinetics, there is XAS-based evidence for sluggish reaction of the Li_2_MnO_3_-type component^25^ and also evidences of a mysterious impedance rise at low SoCs^32–34^ that encourage us to examine whether this is actually because of anionic redox.

Toward these goals, we report here the complete charge-compensation mechanism in LR-NMC as deduced by bulk-sensitive synchrotron-based spectroscopic techniques, namely HAXPES and soft-XAS. The ability of HAXPES to systematically increase probe depths^35^ allowed us to distinguish between surface and bulk effects. We consequently correlate these findings with detailed electrochemical measurements to reveal the interplay between anionic/cationic redox and electrochemical kinetics/thermodynamics of these electrodes. We provide direct evidence of bulk anionic redox activity, which compensates for a substantial capacity not just at high, but quite surprisingly at low potentials also. Moreover, we confirm, as previously reported for Li_2_Ru_0.75_Sn_0.25_O_3_,^10^ that the cationic redox from Ni^2+/3+/4+^ and Co^3+/4+^ exhibits fast kinetics and diffusion in comparison to the sluggish anions. On the other hand, Mn is found to be essentially redox-inactive with only a small contribution from Mn^3+/4+^ at the lowest potentials. To effectively convey these findings, the results will be structured in two parts, dealing first with the charge-compensation mechanism followed by a detailed electrochemical investigation.

## Results

### Anionic/cationic charge-compensation mechanism

**Fig. 1:**
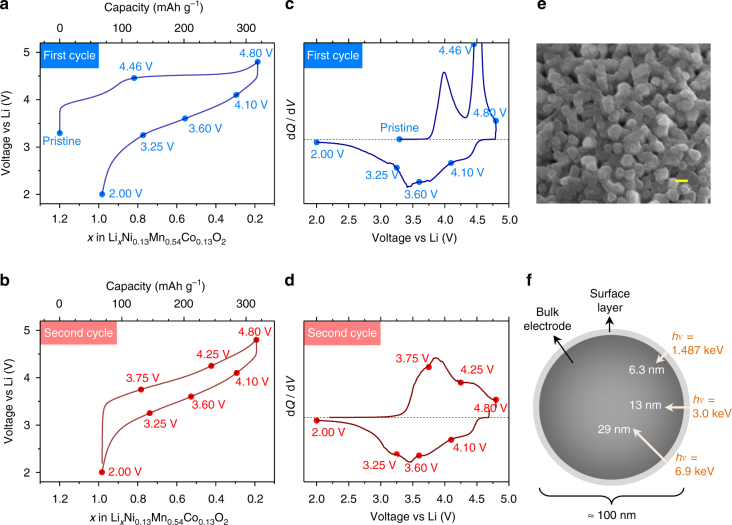
Electrochemical curves with sample positions and HAXPES probe depths. Voltage profiles of Li_1.2_Ni_0.13_Mn_0.54_Co_0.13_O_2_ (LR-NMC) in Li half-cells as a function of Li content with corresponding d*Q*/d*V* curves for the first **a**, **c** and second **b**, **d** cycles. Current density is 20 mA g^−1^. Circles mark the sample positions on the curves. **e** Scanning electron microscopy (SEM) image (scale bar = 100 nm) of the pristine material showing the primary particle size. **f** Schematic view of XPS probe depths estimated for the O 1*s* core peak at increasing photon energies from *hυ* = 1.487 to 3.0 and 6.9 keV. Higher energy photons allow for probing much beyond the surface layer to provide bulk information from the ~100 nm-sized primary particles. The probe depths are defined as three times the photoelectron inelastic mean free path (IMFP) (estimated using the TPP-2M model^36^) that corresponds to 95% of the signal coming from the sample. Note that the probe depths are related to the escape depth of photoelectrons and not to the penetration depth of photons, and that the photoelectron intensity exponentially decreases as a function of the distance from the surface

Li_1.2_Ni_0.13_Mn_0.54_Co_0.13_O_2_ (LR-NMC) powders were prepared by carbonate co-precipitation followed by annealing at 850 °C (see “Methods” section). The obtained single-phase powders were hand mixed with 10% conductive carbon (without binders) and used as positive electrodes in Li half-cells. They exhibit, as widely known, a staircase-like first charge profile that changes to a sloped S-shape on first discharge and this S-shape is maintained from second cycle onward (Fig. 1a, b). During the first two cycles, a series of ex situ samples at different SoCs was prepared by taking apart the cells, then electrode powders being recovered and thoroughly mixed to average-out any concentration gradients. These samples’ SoCs were chosen based on d*Q*/d*V* plots that show several peaks corresponding presumably to different redox couples (Fig. 1c, d). Charge compensation from cationic and anionic redox was examined with XPS performed at increasing probe depths achieved by increasing the photon energy from *hυ* = 1.487 keV (in-house XPS) to *hυ* = 3.0 and 6.9 keV (HAXPES), thus elucidating surface vs. bulk effects. The resulting probe depths^36^ were 6.3 nm at 1.487 keV, 13 nm at 3.0 keV, and 29 nm at 6.9 keV (details of calculation in Supplementary Note 1). Since the LR-NMC primary particles are relatively small (~100 nm, Fig. 1e), the highest photon energy of 6.9 keV can thus access their bulk, as illustrated in Fig. 1f.

Before discussing the spectra, it is worth noting that all precautions have been taken to prevent any contact of the samples with air by maintaining them in dry argon atmosphere or in high vacuum at all steps (details in “Methods” section).

### Anionic redox

**Fig. 2:**
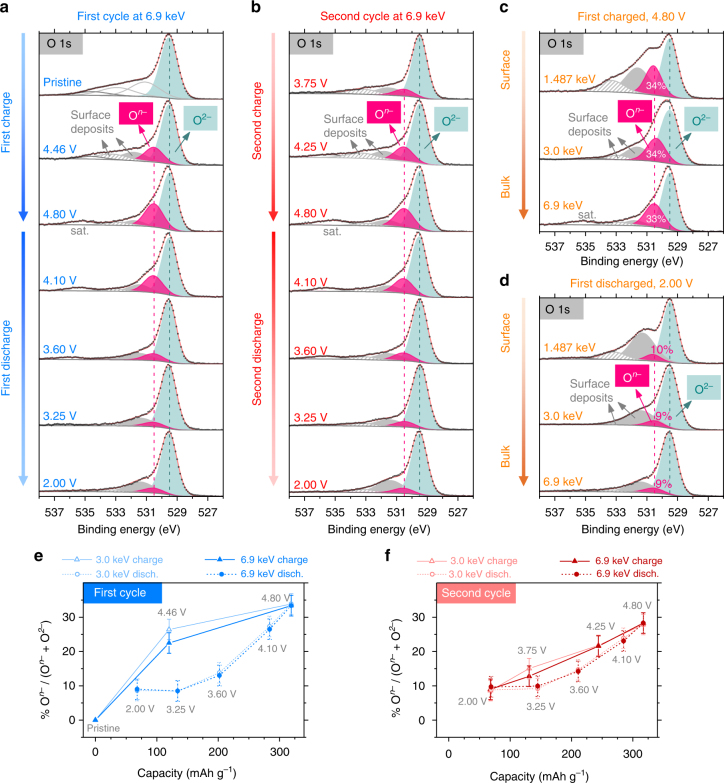
Charge compensation from oxygen (anionic redox) as deduced by HAXPES. Evolution of O 1*s* photoelectron spectra at 6.9 keV (highest probe depth) during the **a** first and **b** second cycles. Black dots are experimental data and red curves are fits. Lattice O^2–^ peak is at binding energy (BE) ≈ 529.5 eV. On charge, oxidized lattice oxygen, O^*n*–^ grows at BE ≈ 530.5 eV (pink peak) followed by its reduction on discharge. Surface deposits appear at higher BEs (gray peaks). Each O 1*s* panel has been normalized to keep the same intensity of the O^2–^ component (green peak) for all spectra. Note that the pristine sample was never in contact with the electrolyte and that its oxygenated surface species are those commonly observed at the surface of oxides. The other samples are electrochemically prepared and hence show new surface oxygenated species compared to the pristine, which come from electrolyte decomposition and passivating surface film formation. An extra satellite peak at BE ≈ 535–536 eV is seen with 6.9 keV (and also weakly with 3.0 keV). The effect of probe depth on the O 1*s* spectra is shown for two samples, **c** first charged at 4.80 V and **d** first discharged at 2.00 V. Surface deposits diminish at high photon energies in contrast to the steady O^*n*–^. Changes in the monochromator energy resolution at different photon energies affect the shape of spectra (the worst energy resolution is obtained for 3.0 keV). Note that each O 1*s* panel has been normalized to keep the same intensity of the O^2–^ component for all spectra. Estimated percentage of oxidized lattice oxygen, % O^*n*–^, is plotted as a function of capacity to understand the anionic charge-compensation during the **e** first and **f** second cycles. Results from 3.0 and 6.9 keV are overlaid in **e** and **f**. The ±3% absolute error bars represent the uncertainty in the fitting procedure, which was determined by comparing the effect of different initial conditions on the resulting fits

Starting with oxygen, Fig. 2a, b, respectively, shows the O 1*s* HAXPES spectra as they evolve during the first and second cycles. For the sake of conciseness, results with only *hυ* = 6.9 keV having the highest probe depth (~29 nm, or 120 times the interlayer distance) are shown here (see Supplementary Figs. 1 and 2 for 3.0 keV and 1.487 keV). The pristine sample is characterized by a strong peak at binding energy (BE) ≈ 529.5 eV due to lattice O^2–^, whereas the smaller peaks at higher BEs come from surface deposits. On charging to 4.8 V, a shoulder grows on the higher BE side of lattice O^2–^ at ~530.5 eV that can be clearly distinguished thanks to the low contribution of surface species and to the very good monochromator energy resolution at 6.9 keV (see fitting procedure details in Supplementary Note 2). The ~530.5 eV BE for this new O 1*s* component is much lower than the values reported for oxygenated species in the surface film. For instance, degradation of carbonate solvents may lead to various organic oxygenated species, but their BEs are observed between 531.5 and 534 eV.^37–39^ Similarly, the inorganic species resulting from degradation of the electrolyte salt LiPF_6_, such as phosphates, fluorophosphates Li_*x*_PO_*y*_F_*z*_ or LiOH, are also observed at higher BEs. Therefore, the ~530.5 eV component can be ascribed to anionic oxygen bound to the TMs. Note, however, the BE increase of +1 eV with respect to classical lattice O^2–^. This can be explained (in light of Extended Hückel Theory-Tight Binding (EHT-TB) and other calculations that have shown a correlation of the BE of oxygen in an oxide with the net charge on the oxygen anion^40–43^) by a significant decrease of the negative charge on oxygen due to its participation in the electrochemical oxidation. The new component at BE ≈ 530.5 eV is thus oxidized lattice oxygen, O^*n*–^ (*n* < 2), which is in complete agreement with our previous report on a series of Li-rich materials (Li_2_RuO_3_, Li_2_IrO_3_, and LR-NMC)^21^ as well as on highly delithiated Li_*x*_CoO_2_ (*x* < 0.4)^44^. Bulk-sensitive HAXPES enables us to unambiguously observe O^*n*–^ on charge and its gradual reduction when discharge proceeds to 2.0 V (Fig. 2a). On further cycling (Fig. 2b), O^*n*–^ regrows at the expense of O^2–^ on second charge and then decreases again on second discharge, thereby confirming the sustained anionic reactivity. An interesting satellite at BE ≈ 535–536 eV is also observed, especially with *hυ* = 6.9 keV. Although the satellite’s origin is still unclear, its gradual energy-shift and intensity variation on charge/discharge suggest its correlation with anionic redox, which should be addressed in future work.

To answer the important question about bulk vs. surface anionic redox, we compare the effect of different probe depths as shown here for two samples, i.e., first charged (Fig. 2c) and first discharged (Fig. 2d). While gradually going deeper into the two samples, the intensity of O^*n*–^ component is not dependent on the photon energy and we do not observe much variation in % O^*n*–^ (percentage of oxidized lattice oxygen), defined as O^*n*–^/(O^*n*–^ + O^2–^) by considering the integrated areas. In contrast, the contribution from surface deposits neatly decreases as *hυ* increases, becoming quite small at 6.9 keV. Thus, we can unambiguously conclude that O^*n*–^ is present in the bulk. Additionally, the quantification of % O^*n*–^ with HAXPES is more reliable than with surface-sensitive in-house XPS because the polluting O 1*s* signal from surface deposits is diminished at high *hυ* and hence the O^*n*–^ peak is not overshadowed.

The overall charge-compensation mechanism from anionic redox can be understood from the changes in % O^*n*–^ as a function of Li insertion/removal. During the first charge (Fig. 2e), % O^*n*–^ grows to 33 ± 3% and then steadily reduces to 9 ± 3% on discharge. O^*n*–^ gradually grows back to 28 ± 3% on second charge (Fig. 2f) followed by its reduction on discharge that is quite similar to the first discharge, hence showing the excellent repeatability of the samples. The ±3% absolute error in % O^*n*–^ quantification (error bars in Fig. 2e, f) arises from the fitting procedure’s uncertainty, which was determined by comparing the effect of different initial conditions on the resulting fits. To this aim, the two surface components were allowed to vary between 531.5–532.0 eV and 533.0–533.5 eV, respectively. These values fall within the range of BEs commonly observed at the surface of various cathode materials cycled with the electrolyte used herein, and are in good agreement with the 1.487 keV spectra (Supplementary Fig. 2) that have higher relative intensities of surface components. Our fitting procedure (details in Supplementary Note 2) aims to prevent over interpretations, but the ±3% absolute error is not insignificant as it leads to rather high relative errors when % O^*n*–^ is low (33% for % O^*n*–^ = 9 ± 3% vs. 10% for % O^*n*–^ = 33 ± 3%) and further work is needed to overcome this uncertainty via improved energy resolution or surface cleaning.

The % O^*n*–^ profiles at 3.0 keV and 6.9 keV neatly superimpose in Fig. 2e, f. However, O^*n*–^ quantification at 1.487 keV is less reliable as stated above, resulting in a slight over prediction of % O^*n*–^ (Supplementary Fig. 3). This underscores the importance of HAXPES in accurately probing bulk anionic redox. The incomplete reduction of oxygen on discharge to 2.0 V suggests its sluggish kinetics and this would also partly contribute to the first cycle irreversibility. A potentiostatic hold at 2.0 V (until current decays to 1 mA g^−1^) is in turn necessary to fully reduce O^*n*–^ (Supplementary Fig. 4), which further strengthens this hypothesis. In light of this result, a detailed study of electrochemical kinetics is presented later. The gradual variation of O^*n*–^ in the second cycle from 2.0 to 4.8 V shows that anionic redox takes place at high as well as at low potentials. This is an interesting result which distinguishes LR-NMC from “model” Li_2_Ru_0.75_Sn_0.25_O_3_ systems in which cationic and anionic redox are well separated at high and low potentials, respectively.^10,45,46^

### Cationic redox

**Fig. 3:**
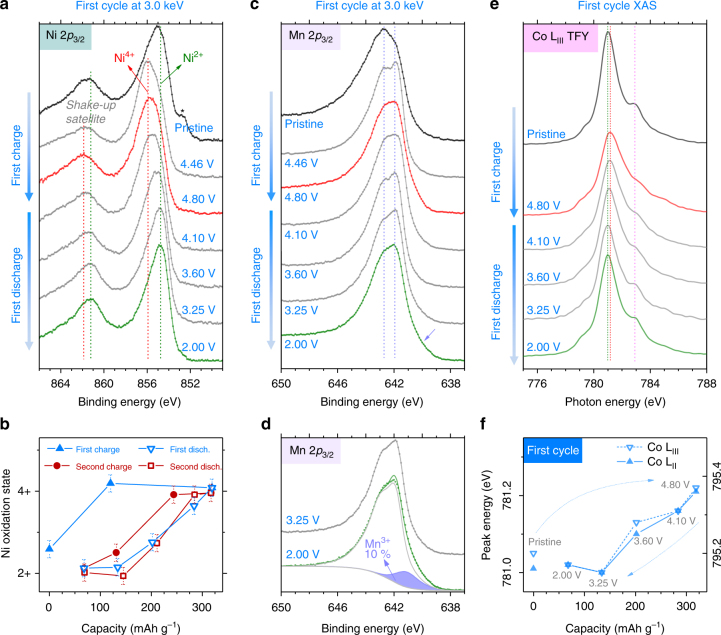
Charge compensation from transition metals (cationic redox) as deduced by HAXPES and XAS. **a** Evolution of Ni 2*p*_3/2_ photoelectron spectra during the first cycle at 3.0 keV (probe depth ≈ 12 nm). The main peak at 855 eV shifts to higher BEs on charge (oxidation from Ni^2+^ to Ni^3+^ to Ni^4+^) and vice versa. A small degradation of the pristine sample under X-ray beam is marked by *. **b** From the BE shift of the main peak, the average Ni oxidation state is estimated as a function of capacity during two cycles. The error bars are determined from the 0.1 eV error in the estimation of the main peak’s maximum. **c** Evolution of Mn 2*p*_3/2_ photoelectron spectra during the first cycle at 3.0 keV (probe depth ≈ 13 nm). The fine structure of the main peak maximum evolves along the two dashed lines. A small shoulder on discharge to 2.00 V (marked by arrow) indicates minor Mn^3+^. **d** The Mn^3+^ amount is estimated by fitting the 2.00 V spectrum with two components, namely the spectrum envelope at 3.25 V (which contains only Mn^4+^) and an additional component (purple peak at ~641 keV) attributed to Mn^3+^. **e** soft-XAS spectra for Co L_III_-edge (dipole allowed transition from 2*p*_3/2_ core level to unoccupied 3*d* states) recorded in TFY mode (probe depth ~100 nm) during the first cycle. The peak slightly shifts to higher photon energy indicating Co oxidation on charge and vice versa. **f** As a function of first cycle capacity, the evolution of Co L_III_ (left) and L_II_ (right) peak energies is shown

Turning now to TMs, their formal oxidation states in pristine LR-NMC are expected as follows: Li_1.2_Ni^2+^_0.13_Mn^4+^_0.54_Co^3+^_0.13_O_2_. Starting with Ni, its 2*p*_3/2_ HAXPES spectra recorded at 3.0 keV during first cycle are shown in Fig. 3a (second cycle is very similar, Supplementary Fig. 5). The Ni 2*p*_3/2_ main peak and its shake-up satellite shift to higher binding energies on oxidation and then come back on reduction. Note that due to the relatively large width of this core peak (full-width half-maximum (FWHM) of ∼3 eV), it is not possible to resolve the underlying Ni^2+^, Ni^3+^, and Ni^4+^ components and only a gradual shift of the whole peak is observed. The average Ni oxidation state can be estimated from the main peak’s maximum position and is plotted in Fig. 3b vs. capacity. The first charge starts with complete Ni oxidation to 4+ before 4.46 V with no further Ni redox during the anionic activation plateau. After first cycle activation, the Ni^2+/3+/4+^ redox process occurs reversibly, but with a noticeable hysteresis loop (Fig. 3b), wherein Ni oxidizes (charge) at lower SoCs compared to Ni reduction (discharge), with the above results in complete agreement with the literature on bulk-sensitive Ni K-edge XAS.^23–26^ This loop is important for voltage hysteresis as we will discuss in detail later. From the variation of Ni oxidation state vs. potential, Ni^2+/3+/4+^ from second cycle onward can be assigned to the same d*Q*/d*V* peak on both charge and discharge, i.e., the middle one around 3.8 V.

Concerning Mn, its 2*p*_3/2_ photoelectron spectra recorded at 3.0 keV during the first cycle are shown in Fig. 3c (second cycle is very similar, Supplementary Fig. 6). The Mn 2*p*_3/2_ peak can be assigned to Mn^4+^ and the fine structure at this peak’s maximum undergoes changes on charge/discharge. Note that the presence of such a fine structure does not indicate two different Mn oxidation states. Other materials containing solely Mn^4+^ are known to show the same kind of Mn 2*p*_3/2_ main peak fine structure, for example, Li_2_MnO_3_, MnO_2_, or Li_2/3_Co_2/3_Mn_1/3_O_2_ (shown in Supplementary Fig. 7). The origin of this particular shape can instead be explained by final state effects in the 2*p* photoemission process involving the first and second neighboring atoms.^47^ Therefore, the evolutions of fine structure in Fig. 3c are not due to changes in Mn oxidation state, but can be attributed to the redox activity of neighboring Ni, Co, and to crystal structure changes. Moreover, the 2*p*_3/2_ fine structure evolves identically when comparing the first and second discharges, thus again demonstrating the sample reproducibility. A slight change in Mn oxidation state occurs at the end of discharge (from 3.25 to 2.00 V), i.e., the appearance of a small shoulder at BE ≈ 641 keV (marked by arrow) denoting partial reduction to Mn^3+^. Mn^3+^ is limited to merely ~10% at the end of first discharge as estimated in Fig. 3d (and ~11% after second discharge, Supplementary Fig. 8). Moreover, Mn^3+^ content is quite similar when characterized with surface-sensitive in-house XPS (Supplementary Fig. 9 and previous study^21^). Therefore, Mn^3+^ reduction is not just a surface effect as widely believed. A potentiostatic hold at 2.0 V (until current decays to 1 mA g^−1^) further increases Mn^3+^ to 18% (Supplementary Fig. 10) confirming the low potential for partial Mn^3+/4+^ activity. Furthermore, this Mn^3+^ is rechargeable, since the Mn^3+^ shoulder disappears in the early stage of second charge (Supplementary Fig. 6). To conclude, the Mn^4+^ state stays mostly unchanged except for a relatively small contribution from reversible Mn^3+/4+^ at low potentials.

Lastly for Co, it is known from previous XPS measurements on layered Li_*x*_CoO_2_ that the oxidation of Co^3+^ does not result in a shift of the Co 2*p*_3/2_ main peak’s position and instead leads to a subtle change in the ratio of the main peak to its shake-up satellite.^44^ As this parameter was not exploitable in our Co 2*p*_3/2_ HAXPES spectra (Supplementary Fig. 11), we performed soft-XAS at Co L-edges (Fig. 3e) in bulk-sensitive total fluorescence yield (TFY) mode. On first charge to 4.80 V, the Co-L_III_ peak shifts to higher energies with a loss of intensity followed by a reverse behavior on discharge to 2.0 V. Likewise, there is also a back-and-forth evolution of the peak shape, especially around 783 eV, where the shoulder is more pronounced for Co in a reduced state (pristine and discharged samples). Similar shifts and shape changes are also observed in the Co-L_II_ peak (Supplementary Fig. 12). Such evolutions in the Co L-edges over charge/discharge are analogous to Li_*x*_CoO_2_,^48,49^ thus confirming some Co^3+/4+^ activity. Complete oxidation of Co to 4+ in these layered oxides is unlikely as it is now well admitted, based on the early prediction of oxygen’s redox participation in Li_0_CoO_2_^50^ that was confirmed later via several characterizations^7^, that O competes with Co for charge compensation at stages of high delithiation. Nevertheless, our spectra demonstrate Co redox activity and to identify its redox potential, the Co-L_III_ and L_II_ peak positions are plotted vs. capacity in Fig. 3f, which indicates a continuous Co reduction down to ~3.25 V. This trend neatly overlays with that of Ni^2+/3+/4+^ from Fig. 3b and therefore, Co^3+/4+^ and Ni^2+/3+/4+^ take place at similar potentials, i.e., as stated above for Ni, around the 3.8 V d*Q*/d*V* peak on both charge and discharge after initial activation.

Altogether, these above-mentioned results clarify the charge-compensation mechanism. The first charge starts with cationic oxidation of Ni and Co (d*Q*/d*V* peak around 4.0 V). Afterward, bulk anionic oxidation, which already begins before 4.46 V, compensates for charge during the activation plateau (d*Q*/d*V* peak around 4.5 V). After first charge, the electrochemistry permanently changes to a sloped S-shape in which Ni^2+/3+/4+^ and Co^3+/4+^ are the main cationic redox processes (maximum 122 mAh g^−1^ assuming the extreme case of complete oxidation to 4+), whereas Mn^4+^ stays essentially inactive except for the minor activity at the lowest potentials. The small amount of Mn^3+^ found at 2.0 V (~11%, compensating for just 19 mAh g^−1^) results from the reversible Mn^3+/4+^ couple, which is known to get activated following the small amount of irreversible oxygen loss on first charge.^15^ Reversible bulk anionic redox, therefore, accounts for a substantial capacity, i.e., at least 109 mAh g^−1^ in 2.0–4.8 V (deduced by subtracting 122 and 19 mAh g^−1^ cationic contributions from 250 mAh g^−1^ total), which is delivered at not just high but quite importantly at low potentials.

### Role of anionic redox on electrochemical properties

A full understanding of charge compensation was necessary to initiate a detailed electrochemical investigation of LR-NMC like we have done for Li_2_Ru_0.75_Sn_0.25_O_3_.^10^ Toward this, we focus on the S-shaped-sloped electrochemistry after first cycle activation, as this is what real-world batteries with Li-rich cathodes will be based on. Activated LR-NMC is afflicted by several performance-limiting issues –namely voltage hysteresis, sluggish kinetics, and voltage fade – and therefore we investigate whether they depend on the anionic/cationic redox interplay.

### Voltage hysteresis

The S-shaped-sloped electrochemistry of LR-NMC is stabilized after a few formation cycles from 2.0 to 4.8 V. Charge–discharge profiles in this wide potential range display a large hysteresis (or path dependence), which is quasi static, i.e., it does not vanish at near-zero currents and is therefore not due to kinetic polarization.^26,28,51–53^ How this hysteresis depends upon the potential cutoffs was tested by progressive opening of voltage windows.

**Fig. 4:**
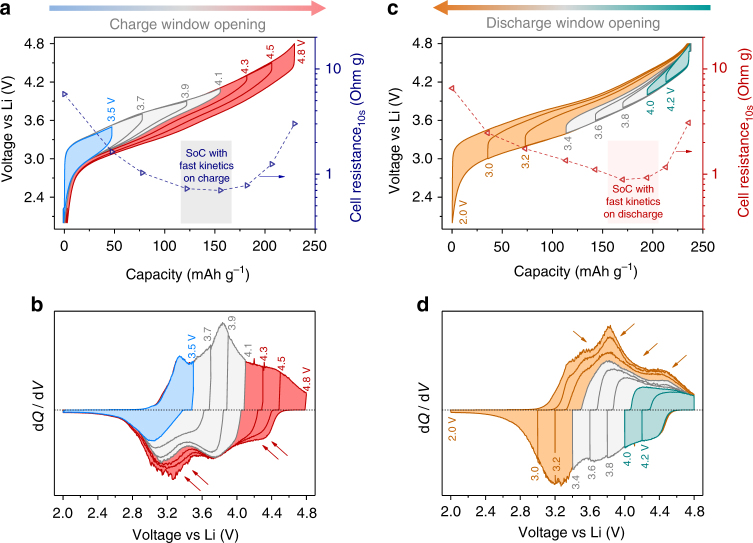
Hysteresis and path dependence in activated LR-NMC studied by voltage window opening. **a** Voltage profiles and **b** corresponding d*Q*/d*V* curves as the charge window is opened stepwise from 2.0 to 4.8 V. LR-NMC is first activated by a few formation cycles in 2.0 to 4.8 V. All curves in **a** and **b** start on the charge direction from 2.0 V (at 0 mAh g^−1^) and trace identical charging paths, whereas the discharges vary with cutoff voltages. Different regimes are highlighted with different colors. Hysteresis is triggered above 4.1 V as the capacity expected from high-potential charge (4.1–4.8 V, red) is split on discharge between high and low potentials, as marked by arrows in **b**. At each cutoff, the cell’s resistance (right axis in **a**) is also estimated by Ohm’s law applied to the voltage relaxation during a 10 s rest-step applied when switching from charge to discharge. Current density is 40 mA g^−1^. The SoC is highlighted, where resistance minimizes during charge. **c** Voltage profiles and **d** corresponding d*Q*/d*V* curves as the discharge window is opened stepwise from 4.8 to 2.0 V, in a fashion opposite the first experiment. All curves in **c** and **d** start on the discharge direction from 4.8 V (fully charged initially). The capacity expected from low potential discharge (3.4–2.0 V, orange) is spread from low to high potentials on charge, as marked by arrows in **d**. The cell resistance (right axis in **c**) is estimated from the voltage relaxation during a 10 s rest-step applied when switching from discharge to charge. The SoC with cell resistance minimum on discharge path is shaded

First, the charge voltage window is opened gradually (Fig. 4a) starting each scan from 2.0 V. Charging beyond 4.1 V (red curves) leads to a noticeable hysteresis that lowers the discharge potential around mid-SoCs. The corresponding d*Q*/d*V* curves reveal that the oxidative capacity above 4.1 V (Fig. 4b, area in red) is only partially recovered on reduction at high potential (down to ~4.0 V), whereas its remaining reduction happens at a significantly lower potential (below 3.6 V), as pointed by arrows. Since cationic oxidation nearly completes before 4.1 V on charge, the d*Q*/d*V* oxidation peak above 4.1 V is mainly charge compensated by anionic oxidation. Because the reduction of thus formed O^*n*–^ is split between high (down to ~4.0 V) and low potentials (below 3.6 V), this causes voltage hysteresis. Moreover, such a change in the sequence of anionic redox also justifies the hysteresis loop seen with HAXPES for Ni’s oxidation state vs. capacity (Fig. 3b).

Next, the discharge voltage window is sequentially opened starting each scan from 4.8 V (Fig. 4c). It is necessary to discharge below 3.4 V (orange curves) for regaining the higher voltage on charge, as seen in the form of a large hysteresis around mid-SoCs. The corresponding d*Q*/d*V* curves show that the capacity reduced below 3.4 V (Fig. 4d, area in orange) is oxidized throughout charging at low as well as at high potentials (marked by arrows). This d*Q*/d*V* reduction peak below 3.4 V is mainly due to anionic reduction, as also supported by the Mn^3+/4+^ contribution remaining minor. Moreover, as the subsequent oxidation of the discharge capacity below 3.4 V is spread across all potentials from 2.0 to 4.8 V, it agrees with the continuous trend of % O^*n*–^ seen with HAXPES in Fig. 2f.

Overall, since cationic redox has the same d*Q*/d*V* peak on either charge or discharge, i.e., the middle one around 3.8 V, it can be concluded that voltage hysteresis is associated with anionic redox, which takes place asymmetrically between charge and discharge.

### Electrochemical kinetics

**Fig. 5:**
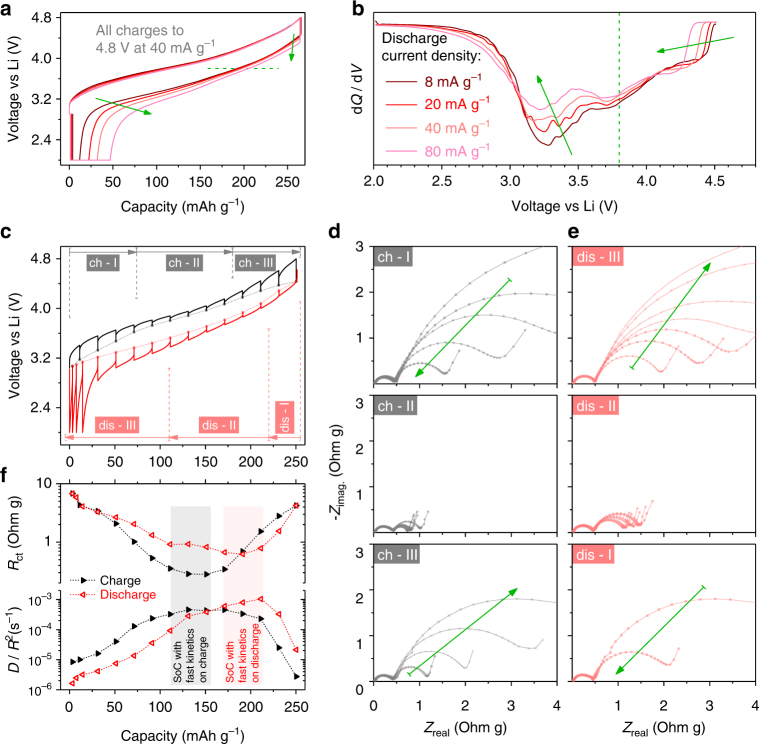
Electrochemical kinetics as a function of SoC for activated LR-NMC. Effect of increasing discharge current densities on **a** half-cell voltage profiles and **b** corresponding d*Q*/d*V* curves. LR-NMC is first activated by a few formation cycles in 2.0–4.8 V. Discharges end with a potentiostatic hold at 2.0 V, until the current decays to 2 mA g^−1^, ensuring identical Li stoichiometry at the beginning of each scan. Arrows show the shift of profiles as current increases. **c** The positive electrode’s voltage profile in a three-electrode cell, recorded with a GITT protocol (40 mA g^−1^ pulses with 4 h rests for equilibration). Circles denoting OCVs are connected with dashed lines to trace a voltage hysteresis loop. Impedance is measured at each step after the 4 h rest. For convenience, charge (black) and discharge (red) are divided into three segments each. During each segment, the evolutions of positive-electrode EIS Nyquist plots are shown for **d** charge (black) and **e** discharge (red). **f** The variation of charge-transfer resistance and diffusion coefficient as a function of SoC on charge and discharge paths. The equivalent circuit model used is shown in Supplementary Fig. 14. *R*_ct_ is the charge-transfer resistance and *D*/*R* ^2^ is the reciprocal of time constant associated with the Li diffusion process, where *D* is the Li diffusion coefficient and *R* is the particle radius. The SoCs with fastest kinetics (minimum *R*_ct_ and maximum *D*/*R* ^2^) are different on charge and discharge paths, as shaded in **f** with black (charge) and red (discharge), respectively

As function of capacity, cell resistance (deduced from the 10 s relaxation steps) displays different trends on charge vs. discharge paths (Fig. 4a, c, respectively), thus again emphasizing a path dependence. Resistance is quite large at low and high SoCs (notice the log scale) and undergoes minima in the middle that are situated at lower SoCs during oxidation in comparison to reduction. Interestingly, the SoCs with minimum resistance correspond to cationic redox around 3.8 V during both charge and discharge. Next, the kinetics of different d*Q*/d*V* peaks was studied by recording the discharge profiles at various current densities (Fig. 5a). On discharging from 4.8 V, the first d*Q*/d*V* peak (above 4.0 V) and the last one (below 3.6 V) are clearly sensitive to changes in current (Fig. 5b), indicating the poor kinetics of anionic redox that takes place at these peaks. Furthermore, note that when discharging from 4.2 V instead of 4.8 V (Supplementary Fig. 13), the cationic redox peak at 3.8 V is not affected by current (meaning fast kinetics), unlike the anionic peak below 3.6 V. To further investigate the role of positive electrode in these results, three-electrode electrochemical impedance spectroscopy (EIS) was performed at different SoCs.

Figure 5c shows the voltage profile obtained via a galvanostatic intermittent titration technique (GITT) protocol, which sets different SoCs where EIS is performed. To conveniently present the EIS Nyquist plots, they are separated into three groups each on charge (“ch-I” “ch-II” and “ch-III”) and discharge (“dis-I” “dis-II” and “dis-III”), as demarcated in Fig. 5c, with the changes in impedance during charge and discharge shown, respectively, in Fig. 5d, e. The high-frequency arc (interphase contacts, surface film, etc.) stays constant and relatively small throughout, whereas significant changes take place in the mid-frequency arc (interfacial charge-transfer) and the low-frequency Warburg tail (restricted solid-phase Li diffusion).^54–56^ Impedance decreases in the beginning of charge path (“ch-I”) and remains small in the middle SoCs (“ch-II”), before increasing again at high SoCs (“ch-III”). A similar trend is seen on discharge. In fact, the large impedance rise at the end of discharge explains why O^*n*–^ is not fully reduced at 2.0 V (Fig. 2) thereby needing a potentiostatic hold to recover anionic capacity.

An equivalent circuit model (Supplementary Fig. 14) is used to quantify the charge-transfer resistance, *R*_ct_ and the diffusion coefficient, *D*/*R* ^2^ (Fig. 5f).^57,58^ In agreement with the trend seen above for cell resistance (Fig. 4a, c), electrochemical kinetics is the fastest in the middle (low *R*_ct_ and high *D*/*R* ^2^), however this occurs at lower SoCs on charge and higher SoCs on discharge, a trend which follows the Ni oxidation state hysteresis loop (Fig. 3b). Alternatively, when *R*_ct_ and *D*/*R* ^2^ are visualized vs. potential (Supplementary Fig. 15), fast kinetics corresponds to the same OCVs (around 3.8 V) on both charge and discharge, which falls over the cationic redox potential range. Overall, it can be concluded that cationic redox has faster kinetics than anionic redox, such that moving away from cationic redox into the anionic redox regime toward higher and lower SoCs (or potentials) causes *R*_ct_ to rise by more than one order of magnitude and *D*/*R* ^2^ reduces by more than two orders.

### Voltage fade

**Fig. 6:**
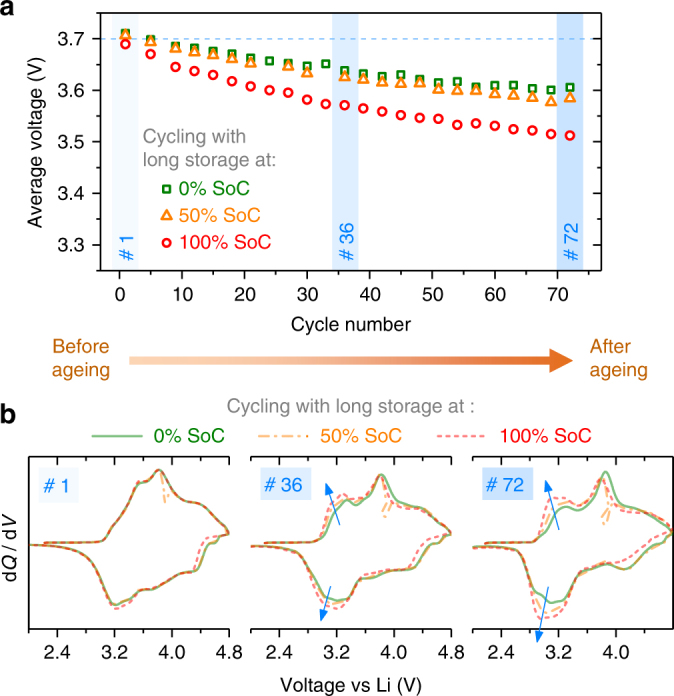
Role of anionic redox on voltage fade during long-term ageing. **a** Effect of storage SoC on voltage fade in a controlled ageing experiment of three LR-NMC half-cells. The cells are identically assembled and activated, after which the same cycling conditions are imposed on the three with only one variable differing, which is the SoC where each cell rests during cycling. Average voltage is defined as the mean of average charge and discharge voltages. Cycling window is 2.0–4.8 V at 40 mA g^−1^ with long (24 h storage) rest periods during every alternate cycle. **b** The corresponding d*Q*/d*V* profiles of the above test in the beginning (cycle #1), mid (#36), and end (#72) of ageing. Arrows show the ageing-related growth of low potential d*Q*/d*V* peaks. One set of curves has small distortions on charge because of this cell’s resting step at 3.9 V (50% SoC)

To study the effects of long-term cycling on voltage fade, a controlled ageing experiment was performed. Three identical half-cells, after a few initial formation cycles, were charged and discharged between 2.0 and 4.8 V, but with long storage periods in every alternate cycle (24 h under open circuit). Everything else being constant, the only difference between the three cells was the SoC at which this storage step was repeatedly performed, i.e., the three cells were cycled with 24 h storage either at 0, 50, or 100% SoC, respectively. Not surprisingly, all three cells show a fade in average cell voltage, defined as the mean of average charge and discharge voltages (Fig. 6a). However, voltage fade is the most severe for the cell that spends its storage periods while being fully charged, i.e., with fully oxidized oxygen. Consequently, the d*Q*/d*V* profiles (Fig. 6b) that are initially identical (cycle #1), start deviating as cycling proceeds. The growth of low-potential redox peaks around 3.0 V can be seen in the mid of ageing (#36) and quite prominently at the end of the experiment after several cycles (#72) for the cell with storage at 100% SoC. We also monitored the impedance growth with ageing that takes place in all cells. However, there is no detrimental effect of storage at 100% SoC, unlike what is shown above for voltage fade. Overall, this ageing study concludes that spending more time in fully charged state, i.e., with highly oxidized oxygen, promotes voltage fade but does not particularly affect impedance growth, which rather appears to be a function of number of cycles.

## Discussion

Via detailed spectroscopic and electrochemical analyses of LR-NMC cathodes, we have revealed (i) their charge-compensation mechanism from anionic/cationic redox, and (ii) how the interplay between these two processes governs application-wise important challenges, such as kinetics, hysteresis, and voltage fade. Next, we connect our results with published knowledge in this area.

The two-stepped first charge voltage profile, typical for Li-rich cathodes, starts with a classical cationic redox step up to ~4.4 V, which is fully reversible^26,28,51,59,60^, followed by the 4.5 V activation plateau where lattice O^2–^ oxidizes to O^*n*–^ (*n* < 2). We have unambiguously spotted O^*n*–^ in the bulk thanks to the high probe depth of HAXPES (~29 nm, 120 layers). HAXPES characterization of anionic redox is repeatable, quantitative, and tunable from surface to bulk, which makes this technique indispensable for studying charge-compensation mechanisms, especially if operando mode is developed.^35^

A simple charge balance suggests that *n* = 1.24 ± 0.07 in the charged sample for 33 ± 3% O^*n*–^ to compensate for the capacity of 0.5 e^–^ per formula unit (157 mAh g^−1^) that was proposed by Luo et al.^4^ This value of *n* is clearly insufficient to make true peroxide (O_2_)^2–^ and the physio-chemical nature of O^*n*–^ should be investigated on a fundamental basis. On further cycling, HAXPES demonstrated the reversibility of anionic reactivity spread in a wide voltage range from 2.0 to 4.8 V. Although such reactivity is beneficial for capacity enhancement, the preceding anionic activation plateau unavoidably causes (i) some irreversible oxygen loss^4,15,16^ and (ii) permanently modifies the electrochemical profile, likely due to oxygen network distortion and rearrangement that is challenging to experimentally visualize. Therefore, we recommend designing cathodes showing a complete reversibility and stability of anionic redox from the very beginning—a task that is theoretically difficult with 3*d* TM-layered oxides.

**Fig. 7:**
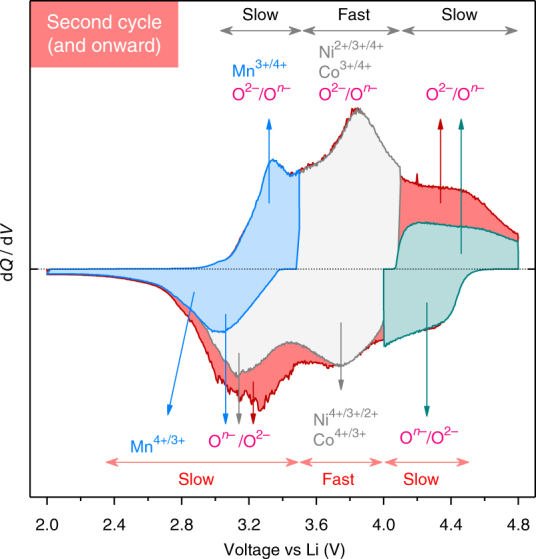
Summary of activated LR-NMC’s charge-compensation mechanism and electrochemical kinetics. Key curves from voltage window opening experiments in Fig. 4 are superimposed on each other, keeping the same colors as before. Electrochemical kinetics is classified as either slow or fast based on impedance analysis in Fig. 5. The small Mn^3+/4+^ contribution is restricted to low potentials (shaded blue) on charge with some anionic activity also. Further charge to 4.1 V (shaded gray) leads to the peak at 3.8 V mainly from cationic oxidation (Ni^2+/3+/4+^ and Co^3+/4+^) along with some anionic contribution. If charging is limited to 4.1 V, the discharge curve (shaded gray) shows two peaks, respectively, due to cationic (3.8 V peak) and anionic (3.2 V peak) reductions. If charging is continued to 4.8 V (shaded red and green), it is mainly charge compensated by anions and then the corresponding discharge capacity is split at high potential (shaded green) and low potential (3.2 V reduction peak, shaded red), thus causing hysteresis. Fast kinetics accompanies cationic redox on either charge or discharge

LR-NMC’s S-shaped-sloped electrochemistry after first cycle activation consists of several d*Q*/d*V* peaks, which we have now assigned properly by combining spectroscopic and electrochemical evidences, as summarized in Fig. 7. Cationic redox occurs at nearly the same potentials during either charge or discharge, such that the middle d*Q*/d*V* peak around 3.8 V shows Ni^2+/3+/4+^ and Co^3+/4+^ activity and the lowest potentials have a minor Mn^3+/4+^ capacity. In contrast, anionic redox proceeds asymmetrically between charge and discharge. On oxidation, anionic redox is spread from 2.0 to 4.8 V with a large contribution above 4.1 V. While on discharge, anionic reduction at high potential is small and the remaining reduction occurs at much lower potentials (peak below 3.6 V). This resolves several charge-compensation-related discrepancies in literature, some of which were called for by recent investigations^61,62^ and reviews^8,63^. For example, the first d*Q*/d*V* discharge peak (4.8–4.1 V) is not from Co reduction as many studies assumed, but rather due to anionic redox. Moreover, this peak is also exhibited by Co-free Li-rich compositions^34,52,60,62,64^, which further confirms our assignment. Another example is the d*Q*/d*V* reduction peak below 3.6 V, which is mainly charge compensated by anionic redox and not by Mn^3+/4+^, whose contribution is very small. Nevertheless, follow-up experiments are underway to quantify the evolution of Mn^3+/4+^ contribution over long cycling.

After the first cycle, a large quasi-static hysteresis of ~500 mV is seen even at C/300^53^, which negatively affects energy efficiency and complicates SoC management. It is now clear, as stated above, that the anionic/cationic redox sequence differs on charge vs. discharge, resulting in path dependence and hysteresis. Such hysteresis mechanism contrasts from LiFePO_4_ (non-monotonic equilibrium potential^65^) or conversion electrodes^66^. Consistent with the anionic/cationic sequence hypothesis, our Ni 2*p*_3/2_ HAXPES results show a hysteresis loop in Ni oxidation state vs. capacity (Fig. 3b). However, we could not observe a complementary hysteresis loop for % O^*n*–^ (Fig. 2f) in a sense opposite to that of Ni, as one would expect for charge balancing. Besides, it also remains unanswered why anionic redox occurs in a wide potential range but differently on charge vs. discharge. One possible explanation could be nested in changes of *n* with SoC, implying different types of O^*n*–^ that can only be unambiguously distinguished with very sharp energy resolutions, thus calling for technical improvements on HAXPES. Previous works suggested a structural origin for hysteresis, i.e., a back-and-forth cationic migration resulting in different thermodynamic potentials during Li removal vs. insertion.^31,53,67,68^ Although this hypothesis is valid, it must be recalled that structural distortions and rearrangements are in fact a consequence of instability induced by anionic oxidation. Nevertheless, the hysteresis mechanism poses a fundamental challenge which could greatly benefit from operando investigations as well as phenomenological models.

Apart from hysteresis, poor rate-capability has been another barrier in the success of LR-NMC. Our results highlight the contrasting electrochemical kinetics of cationic and anionic redox, wherein the latter displays sluggish charge-transfer and diffusion; a situation alike the one previously reported for the “model” Li_2_Ru_0.75_Sn_0.25_O_3_ system, where anionic/cationic activities were unambiguously decoupled.^10,46^ Moreover and worth mentioning is LR-NMC’s large impedance growth at high and low SoCs that is disadvantageous, respectively, for fast charging and discharging.^32–34,69^ To counter this, one can hypothesize, besides electrode blends, a new high-capacity cathode in which cationic and anionic redox occur at the same potential, in order to benefit from fast kinetics of the former as well as extra capacity from the latter. One such known model material is currently being investigated.

Lastly for voltage fade, we have systematically revealed the detrimental role of time spent with highly oxidized oxygen. Thus, the stability of O^*n*–^ is very important for preventing permanent reorganizations, such as irreversible cationic migrations, oxygen loss by recombination or nucleophilic attack, and oxygen lattice modification. Low potential anionic redox is exciting by being more robust against voltage fade^70^, and hence could be a new focus for materials design.

In summary, our detailed spectroscopic and electrochemical investigations of LR-NMC have attempted to answer long-standing questions about this practically valuable system. First, bulk reactivity of reversible anionic redox is now confirmed thanks to HAXPES’s deep probe. Moreover, quantitative tracking of anionic/cationic charge-compensation mechanism in LR-NMC has allowed us to fully understand its d*Q*/d*V* curve. Application-wise, anionic redox boosts capacity but unfortunately, it is also associated with hysteresis, poor kinetics, and voltage fade. Quite remarkably, electrochemical investigation of a model Li-rich system, Li_2_Ru_0.75_Sn_0.25_O_3_, had warned about these side-effects^10^, thus emphasizing the added value of model systems in not just revealing fundamental insights, but also inspiring mitigation strategies. Our future direction would therefore be to master holistically the underlying thermodynamics and kinetics of anionic redox by bridging the learning between model and practical systems. Such an approach is essential for taking high-capacity anionic redox cathodes beyond the labs and into the market.

## Methods

### Material synthesis and characterization

LR-NMC powders were synthesized with a two-step process involving carbonate co-precipitation followed by heat treatment. First, stoichiometric Ni-Mn-Co carbonate was co-precipitated from an aqueous solution of transition-metal sulfates by introducing in it an aqueous solution of sodium carbonate (2 M) and ammonia (0.2 M). This was carried out in a controlled manner (pH = 8, *T* = 55 °C, stirring speed = 1000 rpm) by using a continuously stirred tank reactor (Bioflow 320, Eppendorf) to regulate the morphology and homogeneity of particles. The resulting Ni-Mn-Co carbonate powders were heat treated with Li_2_CO_3_ at 850 °C for 12 h to obtain the LR-NMC powders with a primary particle size of ~100 nm as characterized by SEM (FEI Helios NanoLab 650). The crystal structure was confirmed with X-ray diffraction (BRUKER D8 Advance diffractometer with Cu Kα radiation) and the targeted elemental composition was verified with inductively coupled plasma mass spectrometry.

### Ex situ sample preparation

Li half-cells having LR-NMC powders hand mixed (to preserve morphology) with conductive Carbon Super P in a 90:10 mass ratio at the positive electrode (total weight kept ~20 mg for each cell to ensure repeatability) and Li metal foil at the negative electrode were assembled in Swagelock-type cells in an Argon glovebox (O_2_ < 0.1 ppm, H_2_O < 0.1 ppm). Positive and negative electrodes were separated with two layers of Whatman GF/D borosilicate glass-fiber sheets as the separator soaked with an electrolyte—LP100 (Merck) having 1 M LiPF_6_ dissolved in ethylene carbonate:propylene carbonate:dimethyl carbonate in a 1:1:3 weight ratio. All cells are rested for 12 h before testing. Once the desired SoC is achieved using a constant current density of ± 20 mA g^−1^, the Swagelok cells were disassembled in the glovebox carefully (ensuring no short circuiting) and as soon as possible (to prevent self-discharge under open circuit). The positive electrode powders were rinsed thoroughly three times with anhydrous dimethyl carbonate (DMC) to get rid of the electrolyte and soluble surface deposits. DMC was evaporated by leaving the samples in vacuum (using the glovebox antechamber) for at least 1 h. Note that our ex situ electrode samples were recovered as loose powders that were thoroughly mixed. This averaged out any concentration gradients which can bias XPS results if slurry electrodes are used instead.

### Sample handling and transfer

Great attention was paid to preserve the samples from air and moisture exposure during transfer and handling. They were constantly maintained in dry argon atmosphere or in vacuum. For in-house XPS, they were transferred directly from the argon glovebox (O_2_ < 0.1 ppm, H_2_O < 0.1 ppm) connected to the spectrometer. For HAXPES, they were transferred from the on-site argon glovebox to the beamline introduction chamber (that was kept under vacuum) via a specially designed detachable stainless-steel transfer system (“suitcase”). The samples were first sealed in this “suitcase” inside the glovebox and then transferred to the beamline within 15 min. All series of ex situ experiments (in-house XPS, HAXPES and XAS) were performed on the same samples by pasting the powders on carbon tape.

### In-house XPS

XPS measurements were performed with a Kratos Axis Ultra spectrometer, using a focused monochromatized Al Kα radiation (*hυ* = 1486.6 eV). The analyzed area of the samples was 300 μm × 700 μm. Peaks were recorded with constant pass energy of 20 eV. For the Ag 3*d*_5/2_ line, the full-width at half-maximum (FWHM) was 0.58 eV under the recording conditions. No charge neutralization was required and the pressure was maintained around 10^−8^ mbar. The binding energy scale was calibrated from the C 1*s* core peak at 284.4 eV coming from Carbon Super P added to the positive electrode materials.

### HAXPES

HAXPES measurements were carried out at the GALAXIES beamline of SOLEIL synchrotron facility in France. Photon excitation energies of *hυ* = 3.0 and 6.9 keV were obtained from the first- and the third-order reflections of the Si(111) double-crystal monochromator, respectively. Photoelectrons were analyzed by a SCIENTA EW4000 spectrometer, and the obtained energy resolution from the Au Fermi edge was 0.32 eV for 3.0 keV photon energy and 0.14 eV for 6.9 keV photon energy. No charge neutralizer was required, and the analysis chamber pressure was maintained around 10^−8^ mbar during the measurements. Experiments were carried out using the single-bunch mode (lowest synchrotron brilliance) to minimize degradation under the X-ray beam. The binding energy scale was calibrated to match the in-house XPS spectra on the same samples.

### Soft-XAS measurements

XAS spectra at Co L_II,III_ edges were collected on the ANTARES beamline of SOLEIL synchrotron in France. The ex situ cathodes were transferred from an Ar-filled glovebox to the analysis chamber using the same procedure as for HAXPES measurements, in order to avoid air and moisture exposure. Acquisition using a Bruker detector was carried out in TFY mode with about 100 nm probe depth. The radiation was monochromatized using a plane-grating monochromator (PGM), which is characterized by a slit-less entrance and the use of two varied linear spacing gratings with a variable groove depth along the grating lines. All measurements were performed over the range of 765–805 eV with a step size of 0.1 eV. First, a linear background was subtracted from the XAS spectra, and then they were normalized by the integrated area. Lorentzian curves were used to fit the peak positions of the normalized XAS spectra.

### Detailed electrochemical tests

Bellcore-type free-standing positive electrodes were prepared as described previously^10^ having a composition of 73% (by weight) active material, 9% conductive Carbon Super P, and 18% binder—Poly(vinylidene fluoride-co-hexafluoropropylene). Active material loading is ∼6 mg cm^−2^. The Bellcore method leads to a low loading and high porosity, thereby ensuring that the cell’s electrochemical response is dominated by the active material’s properties. Circular electrodes of 0.95 cm^2^ geometric area were punched out. All electrochemical tests were performed with BioLogic potentiostats in coin-type half-cells (except for three-electrode EIS which was performed in Swagelok-type cells) with Li metal foil at the negative electrode. Coin-type half-cells were assembled in an Argon glovebox with one layer of Whatman GF/D borosilicate glass-fiber sheet as the separator soaked with an electrolyte—LP100 (Merck). All cells are rested for 12 h followed by five formation cycles in 2.0–4.8 V (the first cycle is carried out at 20 mA g^−1^ and the next four at 40 mA g^−1^), resulting in the activation of LR-NMC.

### Three-electrode impedance spectroscopy

T-shaped Swagelok-type cells were prepared with Li metal foil as the counter electrode and a small piece of Li metal as the reference electrode that was fixed at the exposed tip of an otherwise enameled thin copper wire (180 μm diameter). In order to avoid impedance distortions, the reference electrode was placed well within the electrode-sandwich formed by the positive and negative electrodes. Activated LR-NMC was used at the positive electrode after retrieving it from a coin-cell, which had undergone five formation cycles as stated above. This was achieved by disassembling the coin-cell (in discharged state) in the glovebox and quickly transferring the electrode to the fresh three-electrode cell. Glass-fiber sheets soaked with LP100 (Merck) were used as separators. After a few hours of rest to allow wetting and a cycle to confirm the expected electrochemical response, EIS measurements were performed at varying levels of SoC achieved using a GITT protocol. A 10 mV wave was applied with frequencies varying from 200 kHz to 1.4 mHz.

### Data availability

The data supporting the findings of this study are available from the authors on reasonable request.

## Electronic supplementary material

Supplementary Information
