# Supplementary material for: Fundamental interplay between anionic/cationic redox governing the kinetics and thermodynamics of lithium-rich cathodes
Source: Nat Commun. 2017 Dec 20;8:2219. doi: 10.1038/s41467-017-02291-9 (PMC5738393; doi:10.1038/s41467-017-02291-9)
Supplement: Supplementary file 1 — Supplementary Information [file 41467_2017_2291_MOESM1_ESM.pdf]

Supplementary Note 1 | Calculation of XPS and HAXPES probe depths

The probe depths are defined as three times the photoelectron inelastic mean free path (IMFP), and were estimated from the TPP-2M model developed by Tanuma *et al*. [1]. This model uses four parameters: (1) the formula molecular weight, (2) the density, (3) the number of valence electrons per formula, (4) the band gap. Given the composition of the material $Li_{1.2}Ni_{0.13}Mn_{0.54}Co_{0.13}O_2$, its molecular weight is 85.29 $g.mol^{-1}$ and the number of valence electrons is 19.45 per formula. The density is 4.18 $g.cm^{-3}$. The band gap is unknown, but this parameter has only little influence on the final result (for example, the calculated probe depth for $h\nu$ = 6900 eV is 29 nm using band gap = 0 eV and 30 nm using band gap = 5 eV). The band gap was fixed to 2 eV for the calculations.

Supplementary Note 2 | Details on the fitting procedure of O 1*s* spectra

The first rule was to use the minimum of components, i.e. an additional component was introduced only if it was not possible to fit the spectrum properly without it.

The second rule was to constrain the positions and widths of the two O 1*s* components which are attributed to oxygenated species from the surface film. These components are well identified since the corresponding chemical species are also detected by other core peaks, namely C 1*s*, P 2*p*, F 1*s* (not shown in the manuscript because of the great number of spectra, but systematically recorded and quantitatively analyzed). Although the composition of the surface film changes as a function of the electrochemical steps, several oxygenated species arising from decomposition of the electrolyte (i.e. carbonate solvents and $LiPF_6$ salt) are always found in large quantities, e.g. Li_2CO_3, phosphates and fluorophosphates. This makes the O 1*s* signal shape of the surface film rather stable, and very similar to the numerous previous XPS studies on a wide variety of positive electrode materials for Li-ion batteries. Therefore these two components were constrained as follows: the first one was allowed to vary between 531.5 and 532.0 eV and the second one was allowed to vary between 533.0 and 533.5 eV. Their width was constrained to be equal, and allowed to vary between 1.5 and 2.0 eV for the photon energy 6.9 keV (this width depends on the monochromator resolution and it was broader for 3.0 keV).

Sometimes a shake-up satellite was observed, depending on the state of charge. This additional peak is observed at a very high binding energy (535-536 eV) and has no impact on the fitting result of the other components.

The two last O 1s components and the most important ones for the present study, i.e. the lattice O^{2-} peak and the oxidized lattice oxygen O^{n-} ($n < 2$) were fitted without any constraints. The results were very homogeneous in the whole series of samples in terms of line widths and relative position of the O^{n-} peak with respect to the O^{2-} peak.

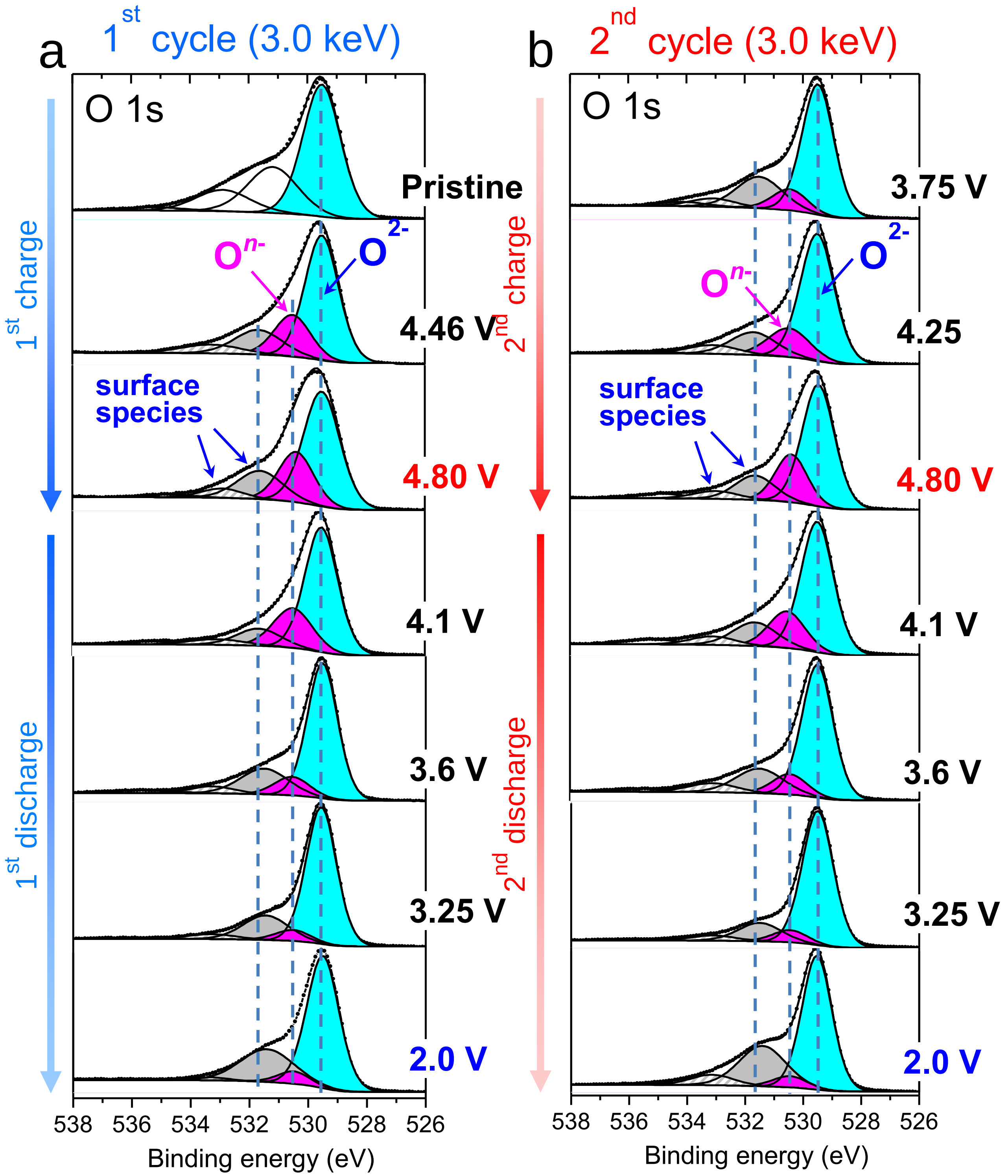

Supplementary Figure 1 | O 1*s* photoelectron spectra of positive electrodes during the (**a**) 1st and (**b**) 2nd cycles recorded with HAXPES at 3.0 keV photon energy.

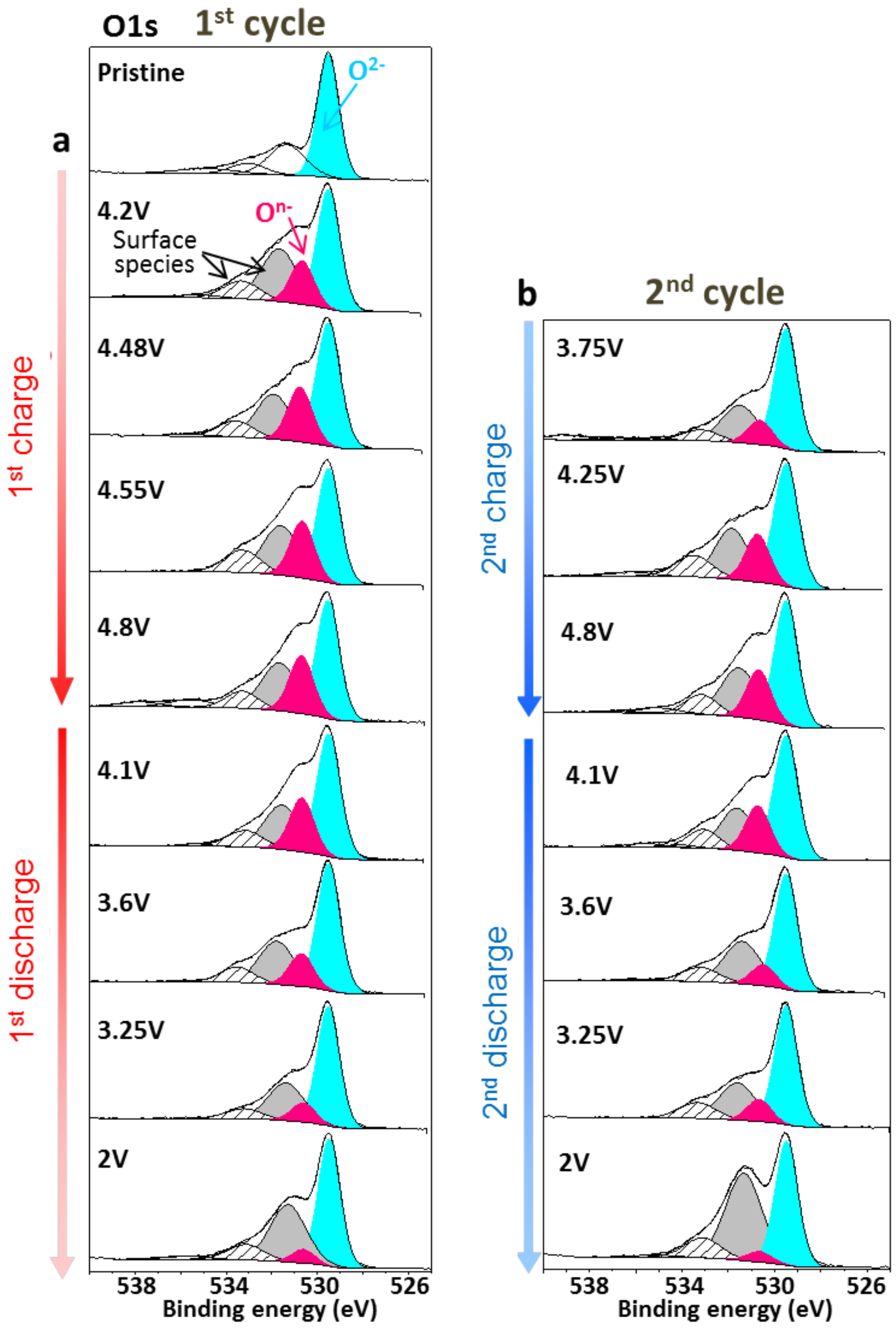

Supplementary Figure 2 | O 1*s* photoelectron spectra of positive electrodes during the (**a**) 1st and (**b**) 2nd cycles at 1.487 keV photon energy (in-house XPS).

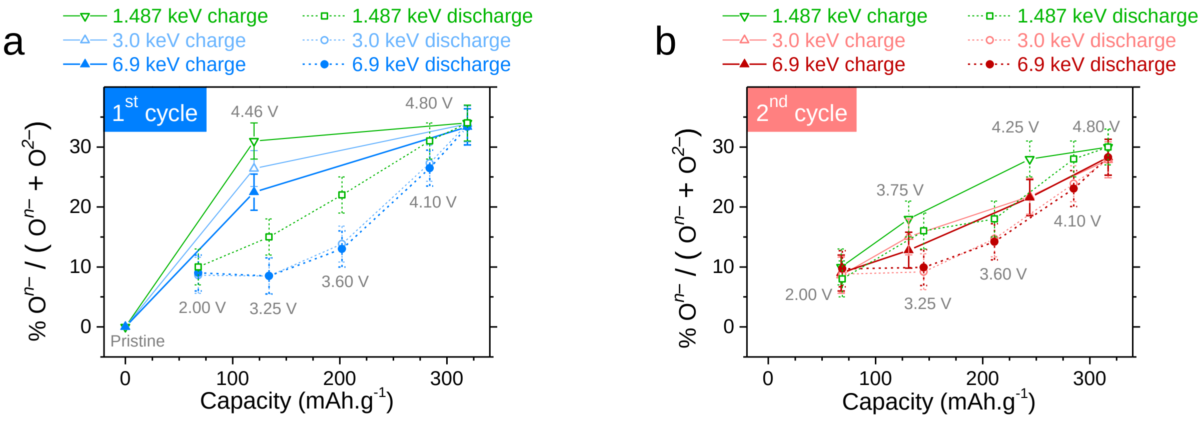

Supplementary Figure 3 | Comparing the effect of increasing photon energies on the estimated percentage of oxidized lattice oxygen in the active materials, defined as O^{n-} / (O^{n-} + O^{2-}), during the (**a**) 1st and (**b**) 2nd cycles plotted as a function of electrochemically observed capacity.

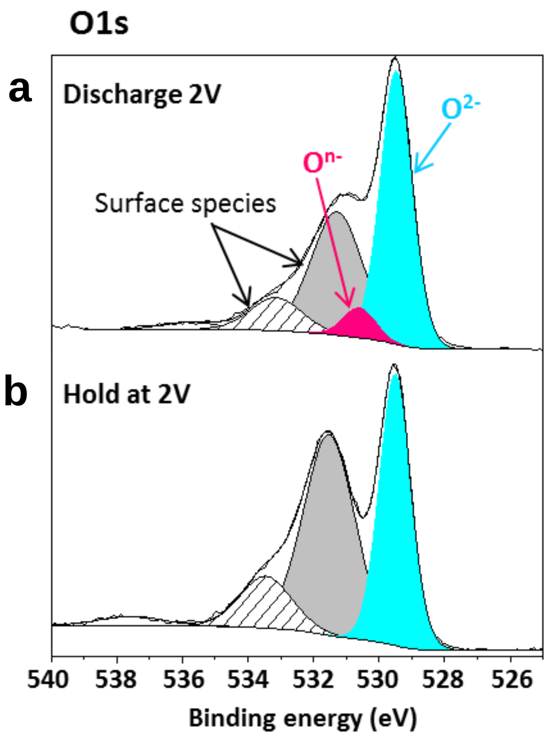

Supplementary Figure 4 | (**a**) O 1*s* photoelectron spectrum of positive electrode after the 1st discharge to 2.0 V using a current density of 20 mA.g^{-1} compared to (**b**) the spectrum after the 1st discharge with similar protocol followed by an additional potentiostatic hold at 2.0 V (until the current decays to a small value of 1 mA.g^{-1}). Photon energy is 1.487 keV.

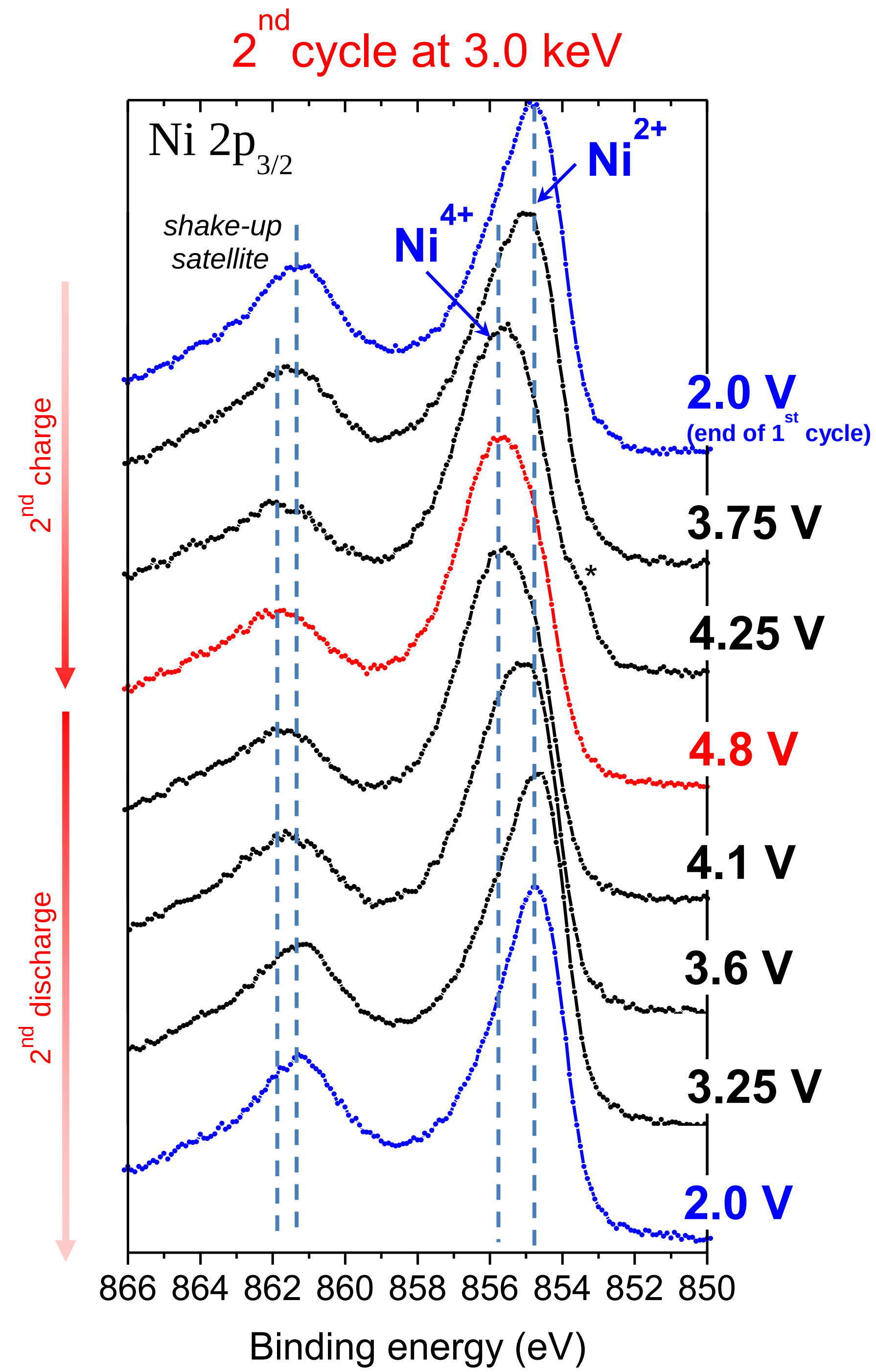

Supplementary Figure 5 | Ni 2$p_{3/2}$ photoelectron spectra of positive electrodes during the 2nd cycle recorded with HAXPES ($h\nu$ = 3.0 keV).

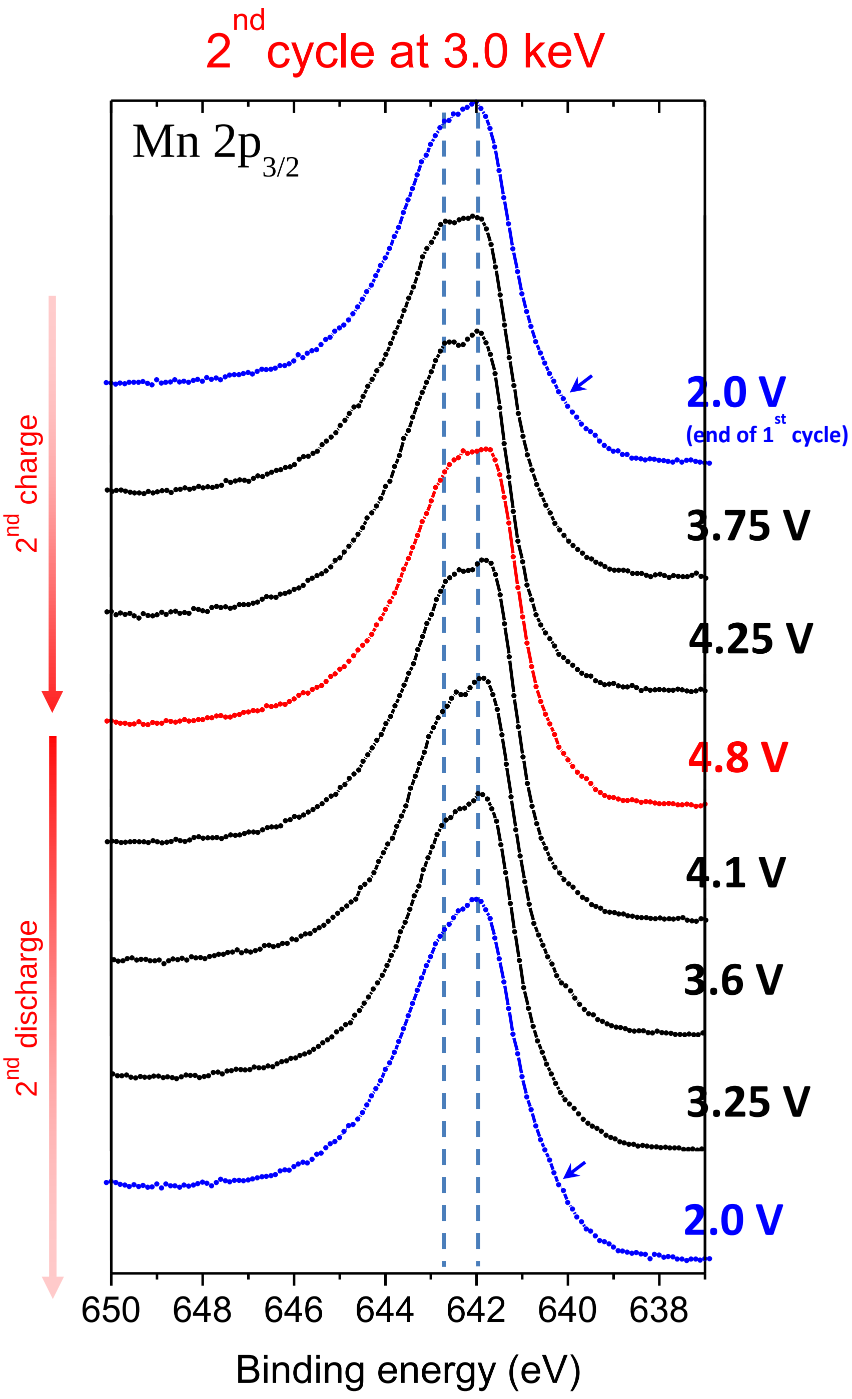

Supplementary Figure 6 | Mn 2$p_{3/2}$ photoelectron spectra of positive electrodes during the 2nd cycle recorded with HAXPES ($h\nu$ = 3.0 keV). Arrows point to the shoulder appearing in the fully discharged samples due to a partial reduction to Mn^{3+}.

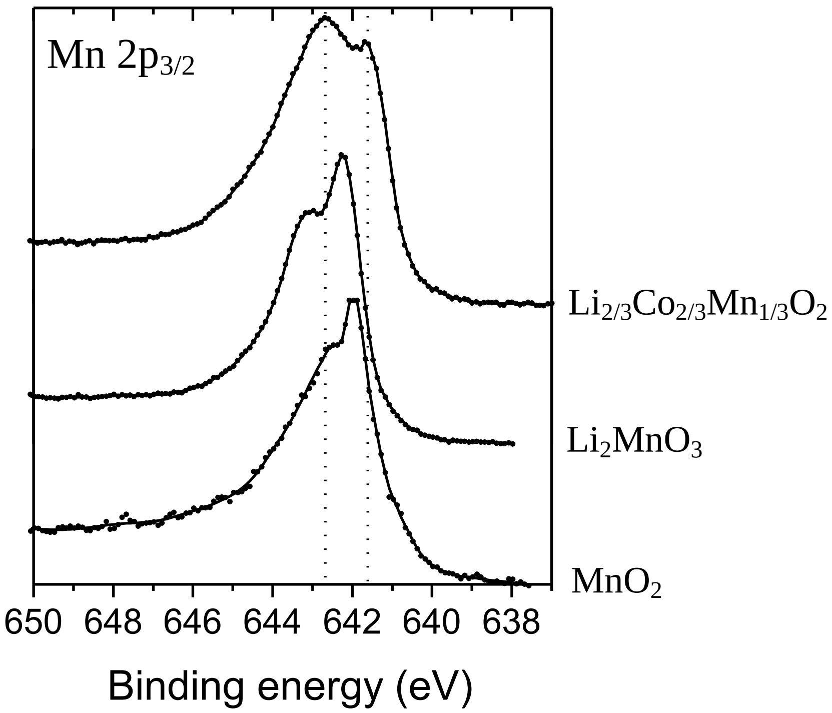

Supplementary Figure 7: Mn $2p_{3/2}$ photoelectron spectra of Mn^{4+} compounds: Li_2MnO_3, MnO_2 and $Li_{2/3}Co_{2/3}Mn_{1/3}O_2$ showing the complex main peak fine structure that cannot be interpreted by two coexisting oxidation states, in the same way as for our Li-rich NMC samples. Spectra recorded at $h\nu$ = 1.487 keV.

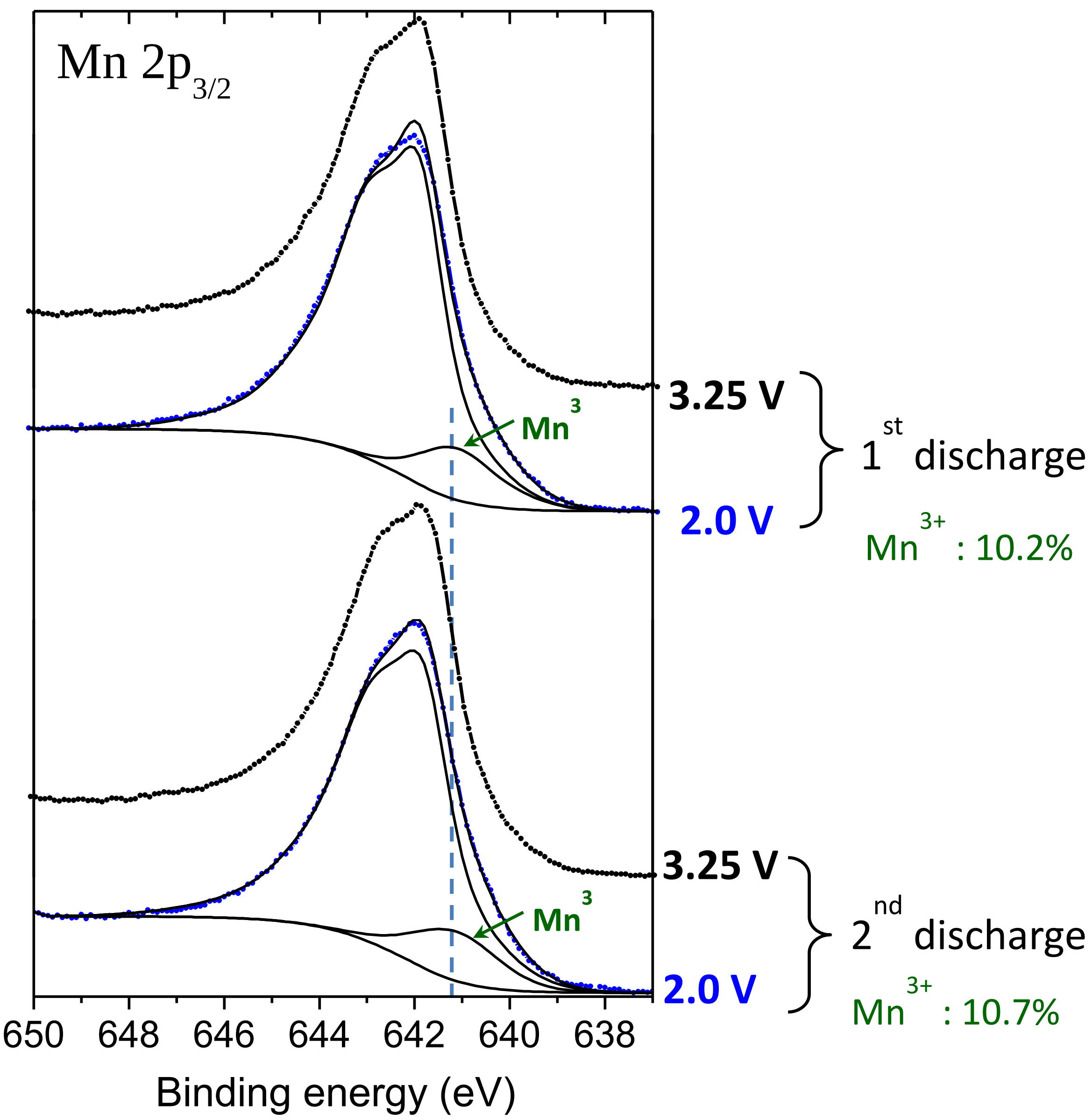

Supplementary Figure 8 | Comparing the amount of Mn^{3+} at the end of the 1st and the 2nd discharges, as estimated from HAXPES spectra (*hν* = 3.0 keV). See main text for details of the fitting procedure.

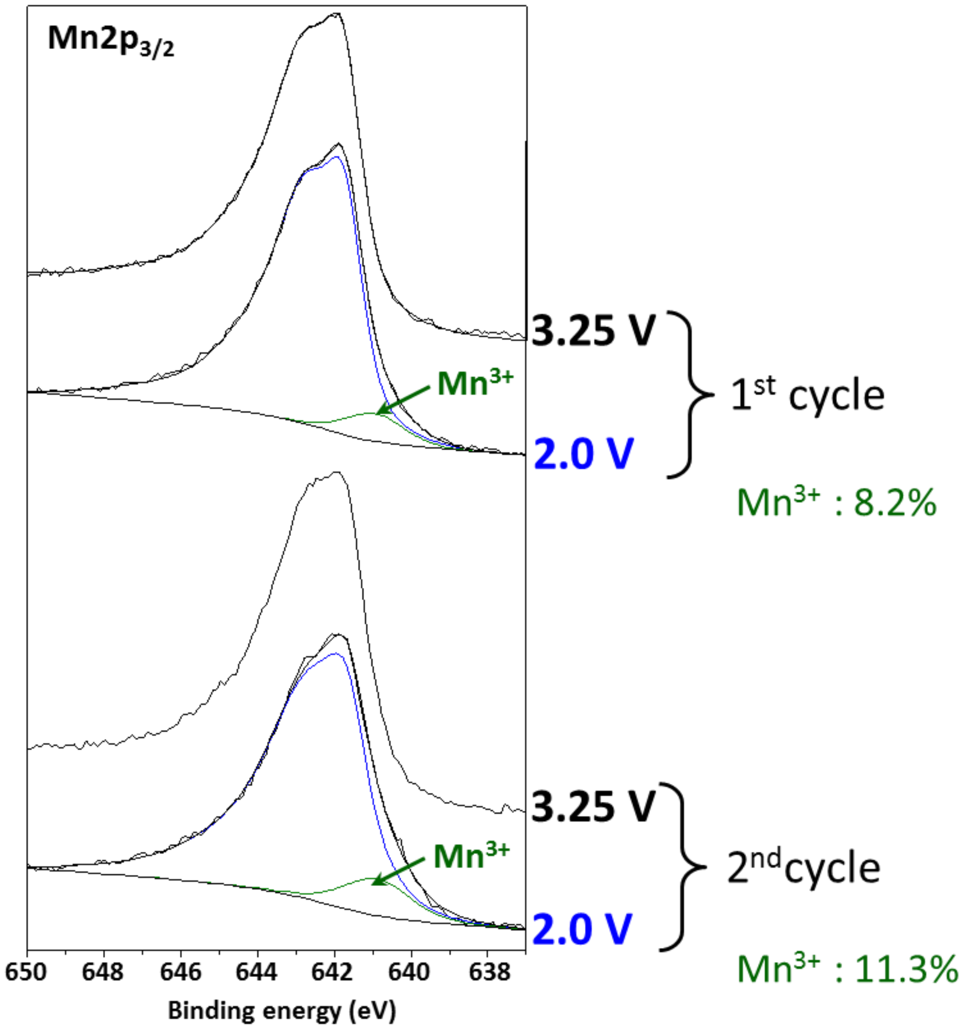

Supplementary Figure 9 | Comparing the amount of Mn^{3+} at the end of the 1st and the 2nd discharges estimated from in-house XPS spectra (*hv* = 1.487 keV).

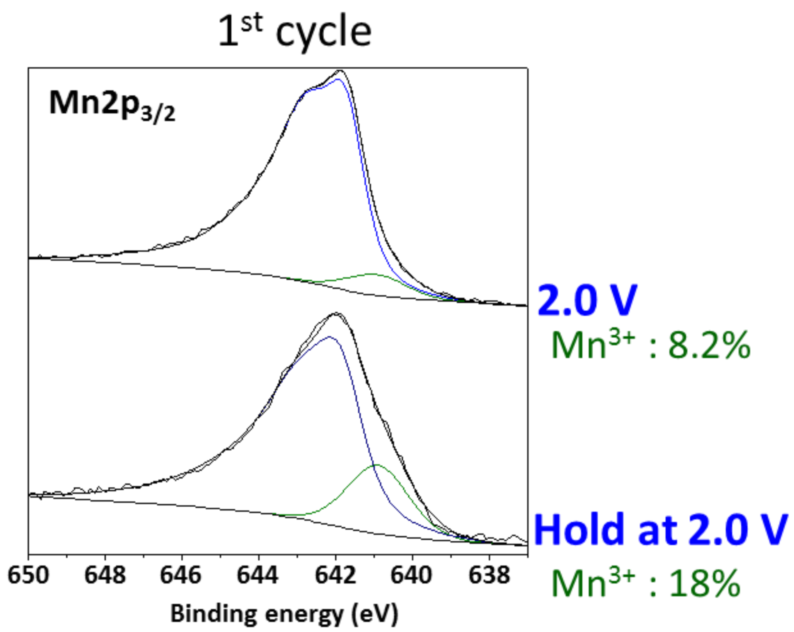

Supplementary Figure 10 | Comparing the amount of Mn^{3+} at the end of 1st discharge with and without a potentiostatic hold. In the top graph, the sample is prepared by discharging till 2.0 V at 20 mA.g^{-1}. In the bottom graph, discharge is continued after reaching 2.0 V by holding the potential till the current decays to a very small value of 1 mA.g^{-1}. The samples are the same as those in Fig. S4. Photon energy is 1.487 keV (in-house XPS).

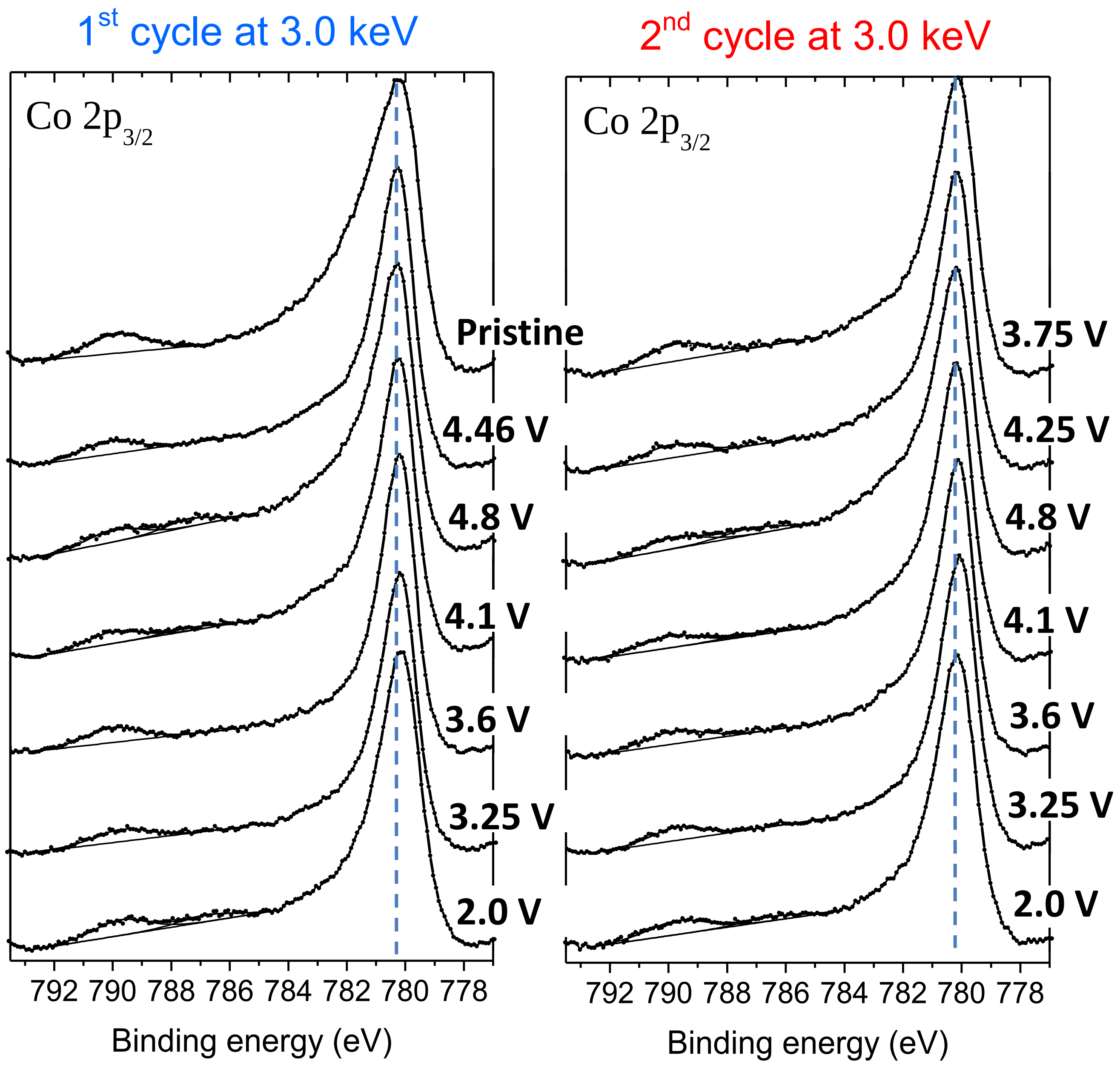

Supplementary Figure 11 | Co 2$p_{3/2}$ photoelectron spectra of positive electrodes during the 1st and 2nd cycles recorded with HAXPES (hv = 3.0 keV). As expected, the main peak of Co 2$p_{3/2}$ does not shift with redox but the area under its shake-up satellite can provide a signature of Co oxidation state change from 3+ to 4+ as previously shown for Li_xCoO_2 [2]. In the beginning of 1st charge, Co oxidizes as evidenced by the loss of satellite intensity from pristine to 4.46 V. Afterwards, it is not possible to quantify clear trends as the satellite is quite small and is further affected by other unexplained satellites due to high photon energy that reproducibly evolve during the 1st and 2nd discharges.

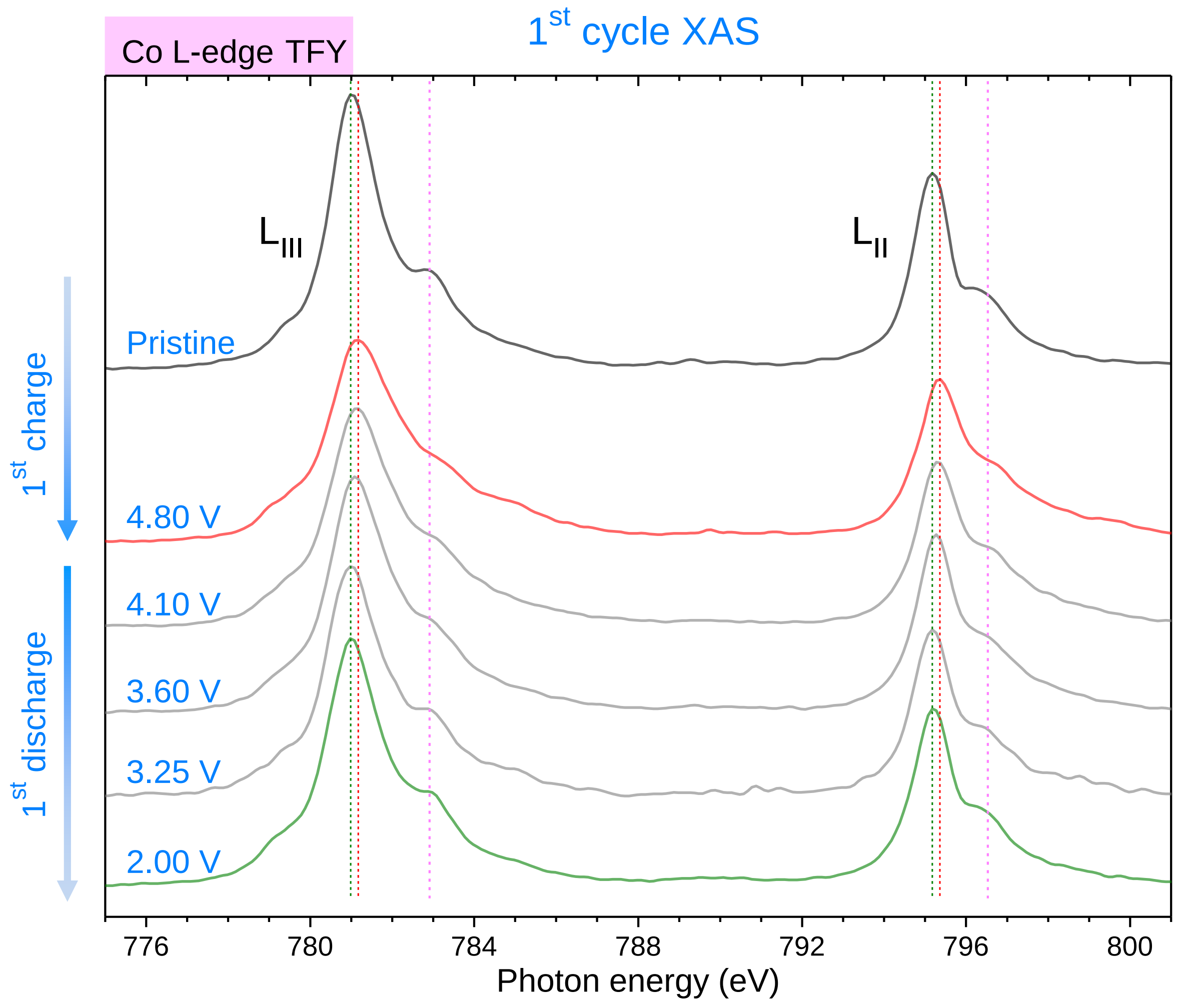

Supplementary Figure 12 | Soft-XAS spectra for Co L-edges recorded in TFY mode during the 1st cycle. Both the L_{II} and L_{III} peaks slightly shift to higher photon energies on Co oxidation (charge) and lower energies on Co reduction (discharge). The peak shapes (shoulders at ~783 eV and ~796.5 eV) also reversibly evolve during charge and discharge.

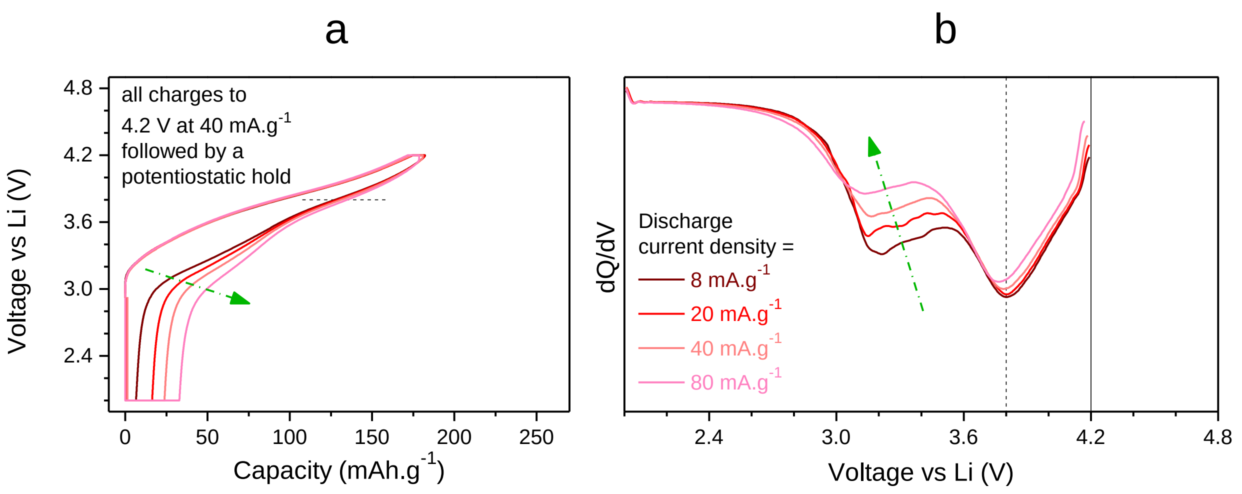

Supplementary Figure 13 | Effect of increasing discharge current densities on (**a**) half -cell voltage profiles and (**b**) corresponding *dQ*/*dV* curves. LR-NMC is first activated by a few formation cycles in 2.0 to 4.8 V. All charges are at 40 $mA.g^{-1}$ till 4.2 V and ending with a potentiostatic hold till current decays to 2 $mA.g^{-1}$ in order to ensure that identical Li stoichiometry is achieved at the beginning of each scan. For the same reason, discharges also end with a potentiostatic hold at 2.0 V, till the current decays to 2 $mA.g^{-1}$. The green arrow indicates the shift in profiles as current increases, thus highlighting resistive behavior.

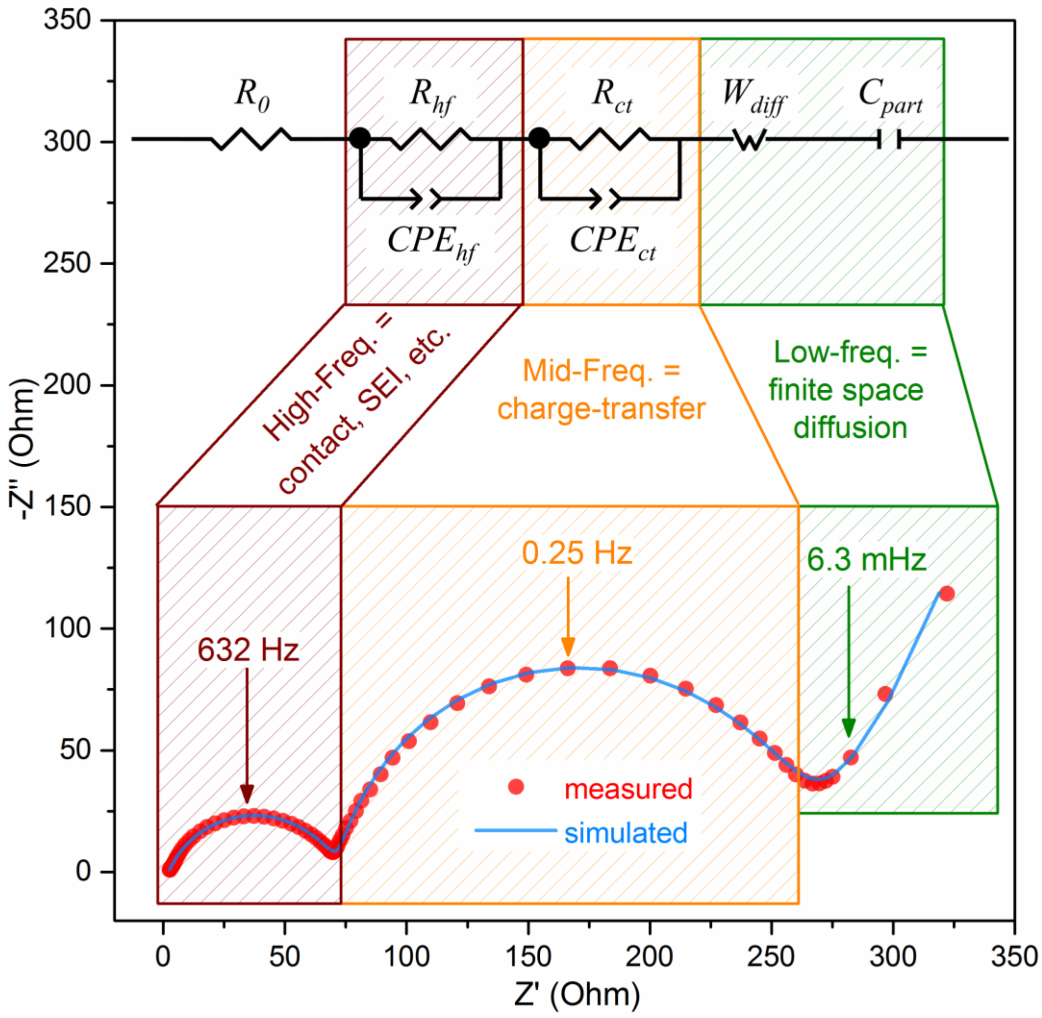

Supplementary Figure 14 | A typical EIS Nyquist plot for the positive-electrode impedance starts with a high-frequency arc representing interphase contacts and surface film etc., followed by a mid-frequency arc signifying interfacial charge-transfer, before ending with a Warburg tail typical of restricted solid-phase Li diffusion [3–5]. The figure shows a good agreement between measurements and simulations based on an equivalent circuit model that is shown on the top part. Features corresponding to different electrochemical phenomena are demarcated and some characteristic frequencies are pointed out. The Fickian diffusion coefficient is calculated by applying a spherical single-particle model using the Warburg resistance obtained at low frequencies, where the imaginary and real parts of impedance show linearity with $f^{-0.5}$, where f is the frequency [5, 6].

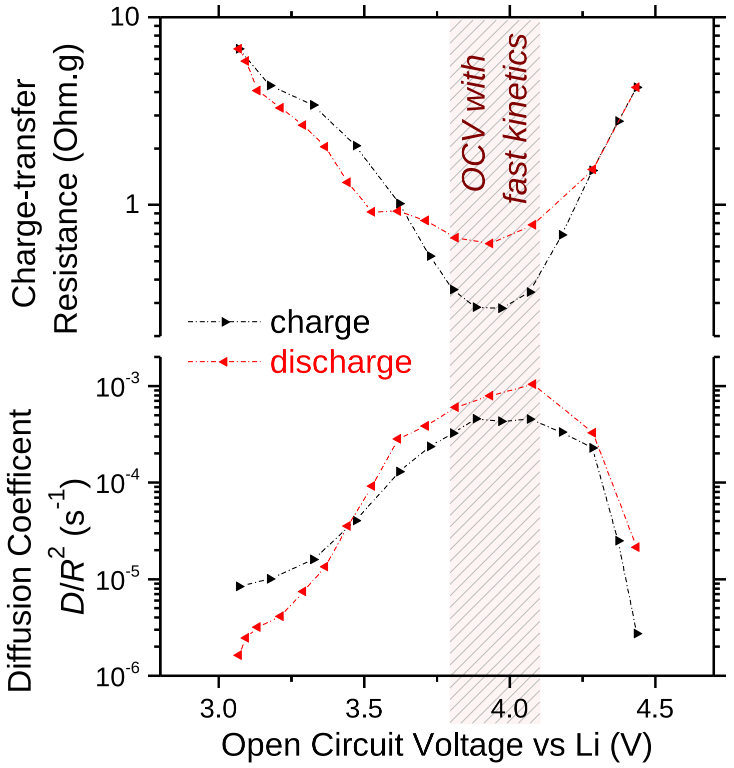

Supplementary Figure 15 | Variation of kinetic parameters obtained from EIS plotted as a function of OCV.

Supplementary References

[1] Tanuma, S., Powell, C. J. & Penn, D. R. Calculations of electron inelastic mean free paths. IX. Data for 41 elemental solids over the 50 eV to 30 keV range. *Surf. Interface Anal.* **43,** 689–713 (2011).

[2] Dahéron, L., Dedryvère, R., Martinez, H., Ménétrier, M., Denage, C., Delmas, C. & Gonbeau, D. Electron Transfer Mechanisms upon Lithium Deintercalation from $LiCoO_2$ to CoO_2 Investigated by XPS. *Chem. Mater.* **20,** 583–590 (2008).

[3] Atebamba, J.-M., Moskon, J., Pejovnik, S. & Gaberscek, M. On the Interpretation of Measured Impedance Spectra of Insertion Cathodes for Lithium-Ion Batteries. *J. Electrochem. Soc.* **157,** A1218 (2010).

[4] Levi, M. D. & Aurbach, D. Simultaneous Measurements and Modeling of the Electrochemical Impedance and the Cyclic Voltammetric Characteristics of Graphite Electrodes Doped with Lithium. *J. Phys. Chem. B* **101,** 4630–4640 (1997).

[5] Ho, C., Raistrick, I. D. & Huggins, R. A. Application of A-C Techniques to the Study of Lithium Diffusion in Tungsten Trioxide Thin Films. *J. Electrochem. Soc.* **127,** 343–350 (1980).

[6] Doyle, M., Meyers, J. P. & Newman, J. Computer simulations of the impedance response of lithium rechargeable batteries. *J. Electrochem. Soc.* **147,** 99–110 (2000).
